# Shape Tailoring of Nonconventional Ni–Pt Nanoparticles through a PDDA-Polyol Method

**DOI:** 10.1021/acsomega.6c03056

**Published:** 2026-09-09

**Authors:** Brayan Maqueda, Ariadna Pérez-Pérez, Alexis Hellmer, Miriam M. Tellez Cruz, Lourdes Bazán-Díaz, Rubén Mendoza-Cruz

**Affiliations:** Instituto de Investigaciones en Materiales, 7180 Universidad Nacional Autónoma de México. Circuito Exterior, Ciudad Universitaria, Ciudad de México 04510, Mexico

## Abstract

Controlling particle morphology is an effective strategy to tune the catalytic behavior of bimetallic nanostructures. Here, we report a simple PDDA-assisted polyol approach to induce systematic shape modulation in nickel–platinum nanostructures. By adjusting the concentration of poly­(diallyldimethylammonium chloride) (PDDA) and the relative Ni/Pt precursor ratio, a gradual morphological evolution was observed, ranging from truncated octahedra to stepped pyramidal and branched flower-like architectures. High-resolution transmission electron microscopy revealed that low PDDA contents promote the development of stepped growth features, whereas increasing PDDA concentrations favor the formation of more complex branched assemblies, consistent with a flocculation-assisted growth pathway. Variations in the Ni/Pt precursor ratio were found to influence compositional trends and surface-related structural characteristics. Catalytic tests using the reduction of 4-nitrophenol as a model reaction showed enhanced activity for these novel nanostructures. Overall, this study highlights the versatility of PDDA as a polymeric growth modulator and emphasizes morphology control as a practical strategy for designing efficient Ni–Pt-based nanocatalysts through a simple wet-chemical approach.

## Graphical abstract

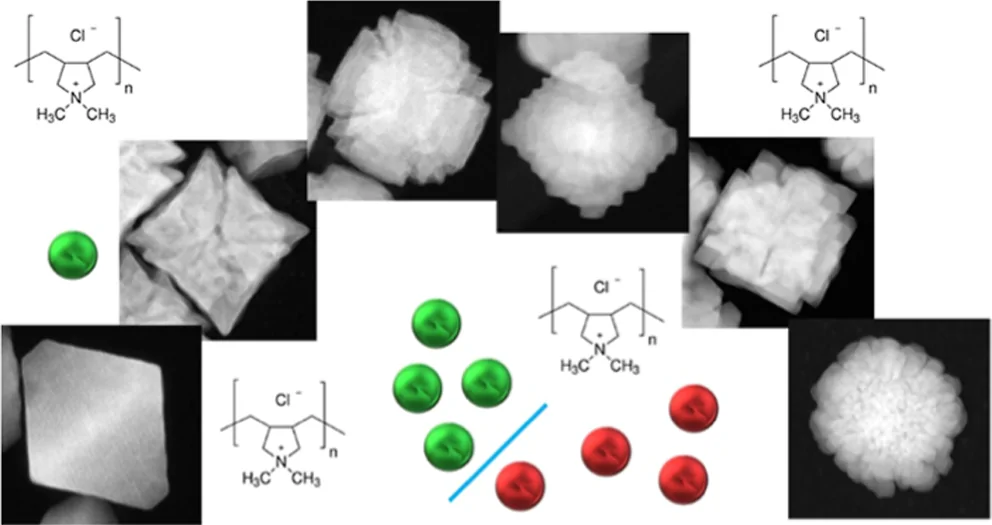

## 1 Introduction

Morphology in nanostructures is a key factor in tuning electronic, structural, and catalytic properties, which depend mainly on the surface atomic arrangement, exposed crystal facets, and structural defect density. It is well established that nanocrystals exposing high-index facets, stepped surfaces, or concave geometries present a larger number of accessible active sites, which often translates into improved catalytic efficiency.^1–4^ Notably, Ni–Pt alloys are particularly interesting due to the combination of platinum’s high catalytic activity and nickel’s electronic tunability, as well as lower cost. Moreover, fine control of size, composition, and facet exposure in Ni–Pt nanoparticles has yielded more active and durable structures.^5,6^

The cationic polyelectrolyte poly­(diallyldimethylammonium chloride) (PDDA) has emerged as a versatile agent for modulating nanoparticle (NP) growth. PDDA can act simultaneously as a stabilizer, a mild reducing agent, and a flocculant. It has been employed to induce unconventional growth pathways, including stepped-pyramidal morphologies in Ni–Pt,^7^ the hydrothermal synthesis of noble-metal nanostructures,^8^ and the self-assembly of hybrid membranes.^9^ Its capacity for layer-by-layer assembly, initially described by Decher,^10^ underscores its relevance in designing functional nano-objects. Additional studies with PDDA–Au systems further highlight its ability to influence optical properties and aggregation behavior.^11,12^ Polymers such as PDDA offer a flexible approach to directing the growth of materials into non-conventional shapes, including truncated octahedra, stepped pyramids, and branched structures. These shapes are designed to enhance performance in model reactions, notably the reduction of 4-nitrophenol.

For Ni–Pt nanoalloys, shape modulation is particularly important because it optimizes the synergy between the two metals, allowing for reduced platinum consumption without sacrificing efficiency. In this context, the reduction of 4-nitrophenol (4-NP) using NaBH_4_ has been widely utilized as a standard reaction to establish clear structure–activity correlations. Its pseudo-first-order kinetics allows meaningful comparison among catalytic systems,^13,14^ and factors such as solvent environment, composition, and surface structure have been shown to affect reaction rates.^15^ In the case of Ni–Pt, enhanced activity has been reported in supported and alloyed catalysts, confirming the synergistic interplay between both metals.^16–18^

In this work, we report a systematic study on the shape modulation of nonconventional Ni–Pt nanoparticles by the PDDA polymer. By varying the PDDA concentration and the Ni/Pt precursor ratio, we obtained a controlled evolution from truncated octahedra to stepped pyramids and branched morphologies. Experimental parameters influencing the final particle morphology are described and the resulting nanostructures were thoroughly characterized, and their catalytic activity was evaluated in the reduction of 4-NP. This report provides insights into the role of PDDA as a growth modulator of Ni/Pt nanoparticles and as a structural variable for designing efficient nanocatalysts.

## 2 Experimental Section

### 2.1 Materials

Ni–Pt nanoparticles (NPs) were synthesized using platinum­(II) acetylacetonate (Pt­(acac)_2_, Aldrich 97%), nickel chloride hexahydrate (NiCl_2_ 6H_2_O, Meyer ACS reagent), poly­(diallyldimethylammonium chloride) solution (PDDA, 20 wt % in H_2_O), ethylene glycol (EG, Meyer, ACS reagent), diethylene glycol (DEG, Sigma Merck), ethanol, and acetone.

### 2.2 Synthesis of Anisotropic Ni–Pt NPs

Nanoparticles with different morphologies were synthesized following the novel protocol described by Bazán-Díaz et al.^7^ The basis synthesis consisted of mixing 20 mL of EG and 1 mL of PDDA into a borosilicate vial. Then, 10 μmol of NiCl_2_ (in 100 μL of DEG) and 10 μmol of Pt­(acac)_2_ (in 100 μL of DEG) were added and homogenized. Then, the mixture was heated to 170 °C (443 K) and allowed to react for 1 h without disturbing. Then, the solution was removed from the hot plate and let to cool down to room temperature. The nanoparticles were recovered by centrifugation at 9700*g* (12,000 rpm) for 10 min. Then, a mixture of ethanol/acetone (1:1) was added to the precipitate, followed by centrifugation and redispersion, repeating these steps three times. The final precipitated NPs were dried and weighed. The basis synthesis was named NiPt:1-1, with the first number denoting the metal precursosr ratio and the second one the PDDA volume (mL). Shape control was achieved in subsequent experiments by systematically varying the PDDA volume and the relative concentration of metal precursors.

### 2.3 Catalytic Reduction of 4-NP

For the catalytic test, NPs were redispersed in distilled water at a concentration of 1.47 mg/mL. The catalytic activity of the Ni–Pt NPs was tested in the 4-NP to 4-aminophenol (4-AP) reduction reaction in the presence of NaBH_4_, as described elsewhere.^7,19^ In a quartz UV cuvette, 250 μL of 4-NP (1 mM) was dispersed in 1.5 mL of distilled water and mixed with 250 μL of NaBH_4_ (300 mM). The 400 nm absorption peak of 4-nitrophenolate was recorded. Then, aliquots of the NP aqueous solution were added into the cuvette, and the optical spectrum was immediately recorded and monitored with time.

### 2.4 Morphological and Structural Characterization

The morphology, crystalline structure, and elemental composition of the produced Ni–Pt NPs weredetermined by high-resolution transmission electron microscopy (HR-TEM), selected area electron diffraction (SAED), and energy-dispersive X-ray spectroscopy (EDS). A JEOL ARM-200F Cs-corrected scanning/transmission electron microscope was used operating at 200 kV and coupled with an Oxford AZtecTEM EDS detector (LUME@IIM-UNAM RRID:SCR_024400). Atomic-resolution images were acquired in the high-angle annular dark-field (HAADF) mode, with convergence and collection angles set to ∼24 mrad and 68-280 mrad, respectively. For 3D tomography, images were collected in a range of −64° to 60°, and the reconstruction was carried out using the Simultaneous iterative reconstruction technique (SIRT). Elemental composition was estimated by inductively coupled plasma mass spectrometry (ICP–MS) using an iCAPQc equipment. UV–vis measurements for catalytic degradation experiments were collected in a Lambda 365+ Perkin Elmer spectrophotometer.

## 3 Results and Discussion

The catalytic behavior of metal nanoparticles depends strongly on their surface and subsurface structure, hence, their activity and efficiency for catalytic reactions can be enhanced by shape variations that lead to changes in exposed crystal facets and coordination environment. The particle shape can be modulated through the selection of different capping agents. Based on the protocol developed by Bazán-Díaz^7^ for the synthesis of non-conventional nanoalloys in EG, herein we finely tuned the shape and structure of Ni–Pt nanoalloys by modifying simple synthesis parameters, namely capping agent and metal precursor concentration, which had a strong repercussion on their final morphology and catalytic activity.^3,4^ A sketch of the employed methodology is presented in the Supporting Information, Figure S1, and a photograph of the distinct colloidal solutions is presented in Figure S2.

### 3.1 Effect of PDDA Concentration

The EG volume was fixed at 20 mL and Pt/Ni ratio was maintained to 1, while the effect of PDDA was studied by varying the volume added during synthesis. The sample ID and experimental parameters are summarized in Table 1, where the notation reflects the Ni/Pt ratio and the PDDA volume (mL). TEM images in Figures 1 and S3 show how particle morphology evolved as a function of the PDDA volume (EG to PDDA ratio from 80 to 4), showing that PDDA strongly influences the formation of surface protuberances. This variation promoted a clear evolution from a quasiregular geometrical shape to a protruding and flower-like shape as the PDDA concentration increased. At low PDDA content (NiPt:1-0.25 and NiPt:1-0.5), the particles predominantly resemble a truncated octahedral shape (TO) with facet reconstruction. More irregular shape and inhomogeneous distribution was present when lower PDDA volumes were used, with the presence of multiply twinned structures, as seen in Figure 1a, which reduced at higher surfactant content. When PDDA increased to 1–2 mL (NiPt:1-1 and NiPt:1-2), a better shape distribution was achieved with a stepped pyramidal growth on the particle faces.

**Table 1:** Experimental Parameters and Chemical Composition Obtained by Changing the Amount of PDDA Used for the Synthesis of Ni–Pt Nanoparticles

sample ID	figure	PDDA (mL)	EG/PDDA	NiCl_2_ (μmol)	Pt_(acac)_ (μmol)	Ni/Pt ratio	Ni at%–Pt at% (EDS)
NiPt:1-0.25	[Fig fig1]a, 2	0.25	80	10	10	1	41.3–58.7
NiPt:1-0.5	[Fig fig1]b, 2a	0.5	40	10	10	1	47.9–52.1
NiPt:1-1	[Fig fig1]c, 2b	1	20	10	10	1	50–50
NiPt:1-2	[Fig fig1]d, 2c	2	10	10	10	1	48–52
NiPt:1-3	[Fig fig1]e	3	6.67	10	10	1	42.7–57.3
NiPt:1-4	[Fig fig1]f, 2d	4	5	10	10	1	33.3–66.7
NiPt:1-5	[Fig fig1]g, 2	5	4	10	10	1	25–75

**Figure 1:**
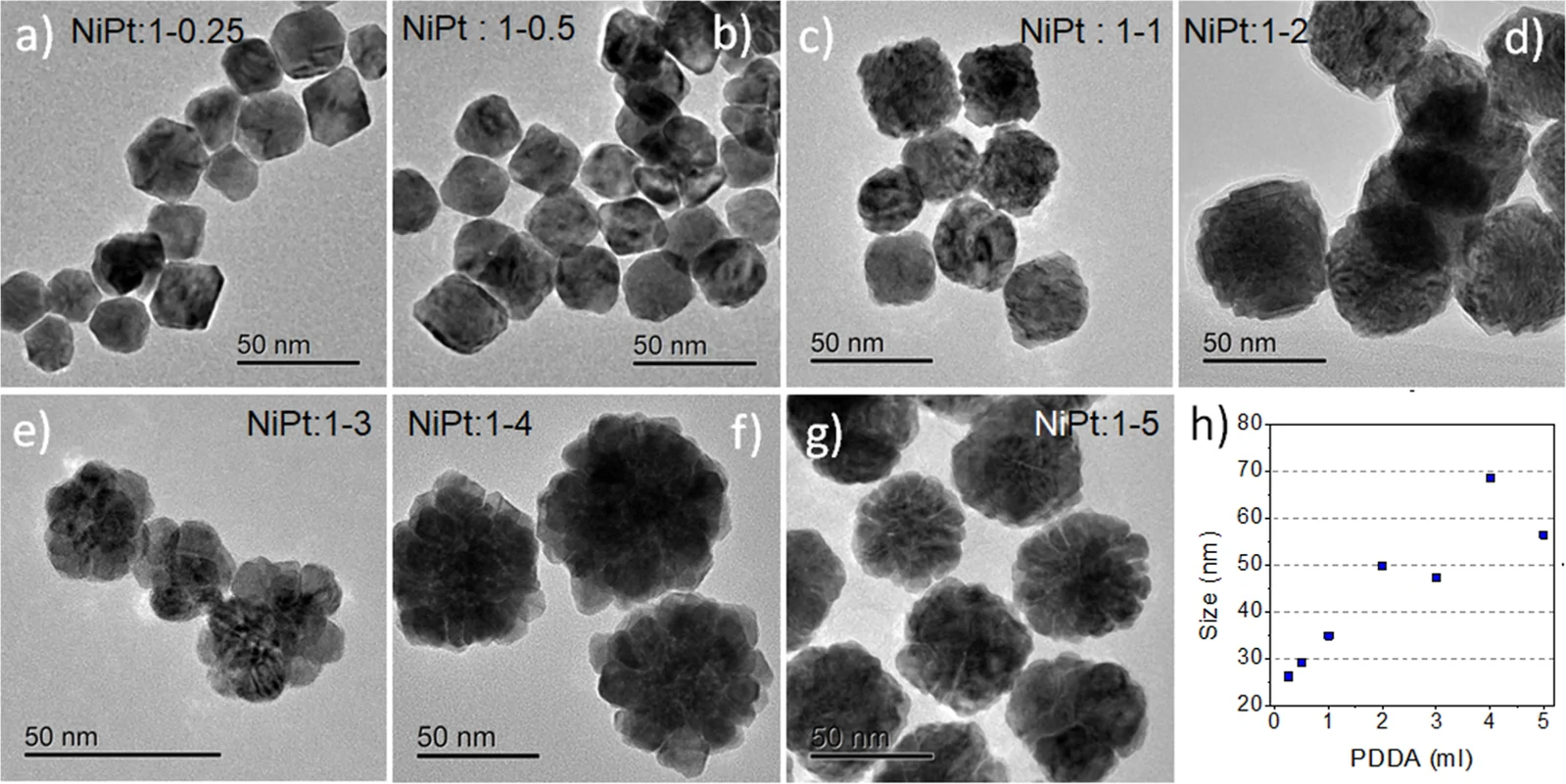
TEM images showing the morphological evolution of Ni–Pt NPs as a function of PDDA volume: (a) 250 μL (NiPt:1-0.25), (b) 500 μL (NiPt:1-0.5), (c) 1 mL (NiPt:1-1), (d) 2 mL (NiPt:1-2), (e) 3 mL (NiPt:1-3), (f) 4 mL (NiPt:1-4), and (g) 5 mL (NiPt:1-5). (h) Particle size as a function of the PDDA volume.

As the PDDA content further increased, a protuberated, multibranched shape was promoted, with an increasing number of branches as PDDA rose from 3 to 5 mL. The branches grew sharper in the range of 3–4 mL, while 5 mL they became smoother and less defined. Moreover, the size distribution slightly broadened as PDDA increased, Figure S4. These results highlight the versatility of the method, demonstrating that a precise shape modulation can be achieved by variation of just one parameter (PDDA volume) with excellent homogeneity, as shown in Figure S3. The average particle size increased progressively with PDDA content (Figures 1h, S4).

HR-TEM and HAADF imaging enabled the clear visualization of the shape tuning and the effect of the surfactant on the crystalline structure of the particles as a function of PDDA volume, Figure 2. Fast Fourier transform (FFT) analysis was performed to reveal the crystalline structure and correlate the viewing directions of the crystal lattices with the particle shape. In the HAADF images, the [001]_FCC_ direction is indicated to highlight the differences in shape and crystal growth. From these analyses, the lattice symmetry and planes were determined, matching the face-centered-cubic (FCC) structure of a single-phase Ni–Pt alloy. Additionally, selected-area electron diffraction (SAED) showed single-phase FCC patterns, confirming the alloyed nature of the particles (Figure S5). At low-PDDA concentrations (NiPt:1-0.25 to NiPt:1-2 samples in Figure 2), both the TO-like and the stepped particles crystallized as single crystals despite the intricate shape. The stepped surfaces observed in NiPt:1-1 and NiPt:1-2 followed the crystalline structure of the core, with PDDA serving as a stepped growth modulator. It is worth noting that the step pyramidal growth reported by Bazan et al.^7^ was achieved with an optimized EG/PDDA ratio of 20 (NiPt:1-1), whereas reducing the ratio to 10 produced a different stepped growth with extended and sharper step edges (NiPt:1-2), revealed from the HAADF images.

**Figure 2:**
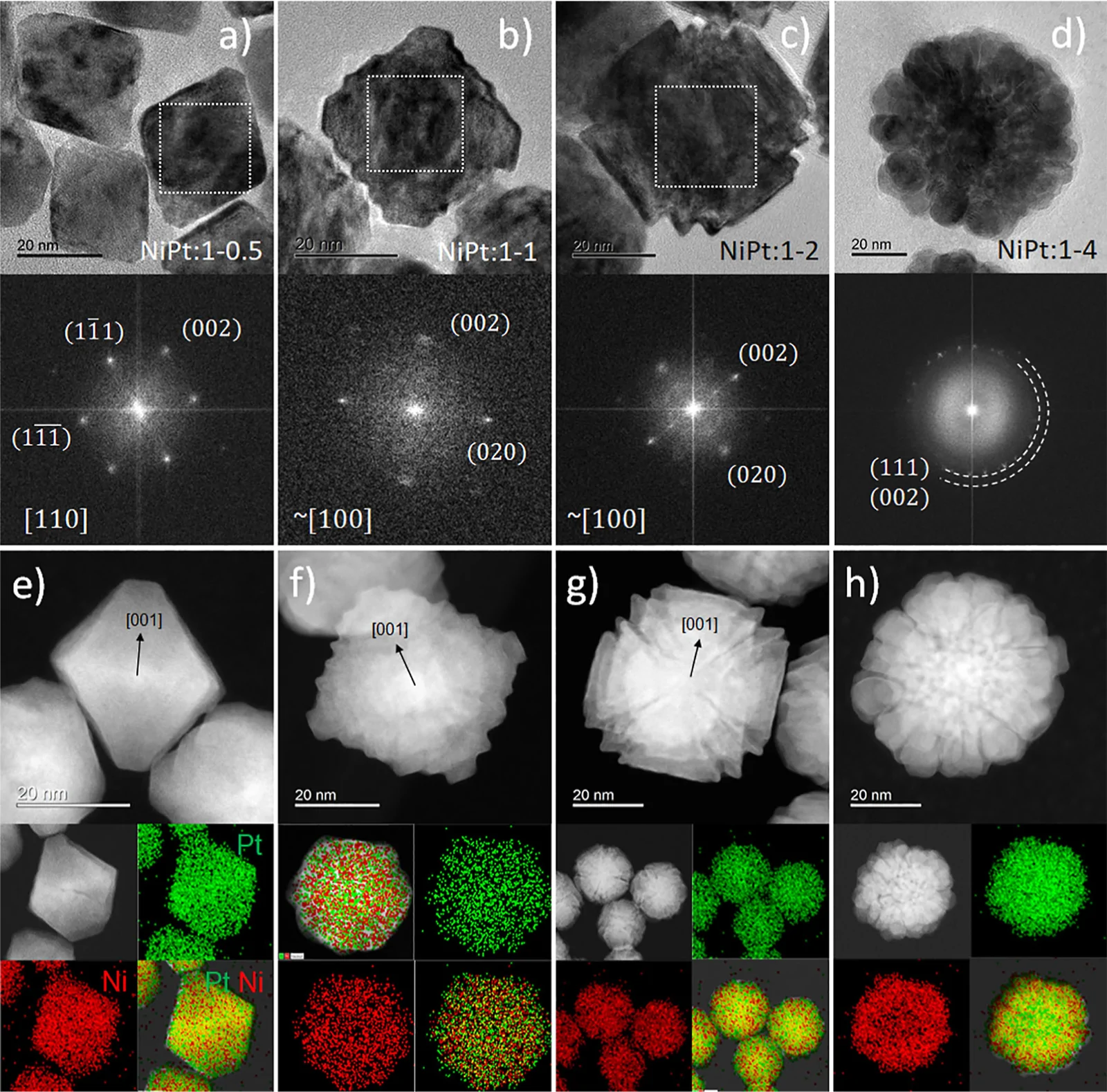
HR-TEM images and their FFT of the indicated areas (top), and HAADF-imaging and EDS mapping (bottom) of the Ni–Pt NPs produced using different PDDA volumes: (a,e) 500 μL (NiPt:1-0.5); (b,f) 1 mL (NiPt:1-1); (c,g) 2 mL (NiPt:1-2); and (d,h) 4 mL (NiPt:1-4).

As the PDDA presence increased, a branched flower-like shape developed with multiple branches and a polycrystalline structure, as observed from the ring pattern-like in the FFT. The branches are formed by small single-crystal particles that agglomerated, forming a complex external shape with an intricate internal channel structure, which increased in complexity with particle size and PDDA content. This behavior is attributed to the role of PDDA as a flocculant agent, previously reported in the synthesis of nanoparticles and the formation of assemblies,^8,9,12,20,21^ where it promotes the agglomeration of small Ni–Pt seeds leading to the final flower-like multibranched morphologies.

The chemical composition of these nanoalloys, as determined by EDS and electron diffraction (Vegard́s Law), also depends on the amount of PDDA present during synthesis (Figure 2e–h; Table S1). As PDDA increased, the nickel content increased for small and medium PDDA concentrations (NiPt:1-0.25 to NiPt:1-2), suggesting that PDDA acts as a mild reducing agent for the Ni^2+^ ions and promotes their incorporation into the nanoalloy structure.^11^ At higher PDDA concentrations, however, Pt reduction was increased, obtaining Pt-rich nanoalloys with intricate morphologies (Table 1). The role of PDDA in promoting the reduction of metal ions can be inferred from the progressive darkening of the reaction solutions, indicating a higher concentration of particles as a function of PDDA (Figure S2).

### 3.2 Effect of Relative Metal Precursors Concentration

The effect of the relative metal precursor concentration was also explored. Two experimental series were carried out, by fixing PDDA volume to either 1 or 2 mL, while varying the ratio between nickel chloride and platinum acetylacetonate. In Figures 3 and S6, TEM images show the obtained particles for the two series. The sample IDs, experimental parameters, and chemical compositions are summarized in Table 2. The change in relative metal proportion also led significant changes in the final morphologies. For the 1 ml PDDA series (Figure 3a–e), the protuberant growth was finely depressed when more nickel chloride was present during the synthesis. Multibranched nanoparticles were obtained when a synthesis was performed with a Pt-rich environment, while more rounded particles were achieved in Ni-rich compositions. The actual chemical composition of these particles followed the nominal metal concentrations, with Pt at 90–50%. However, in the Ni-rich range, the chemical composition showed a nickel deficit in the particle structure (Table 2).

**Figure 3:**
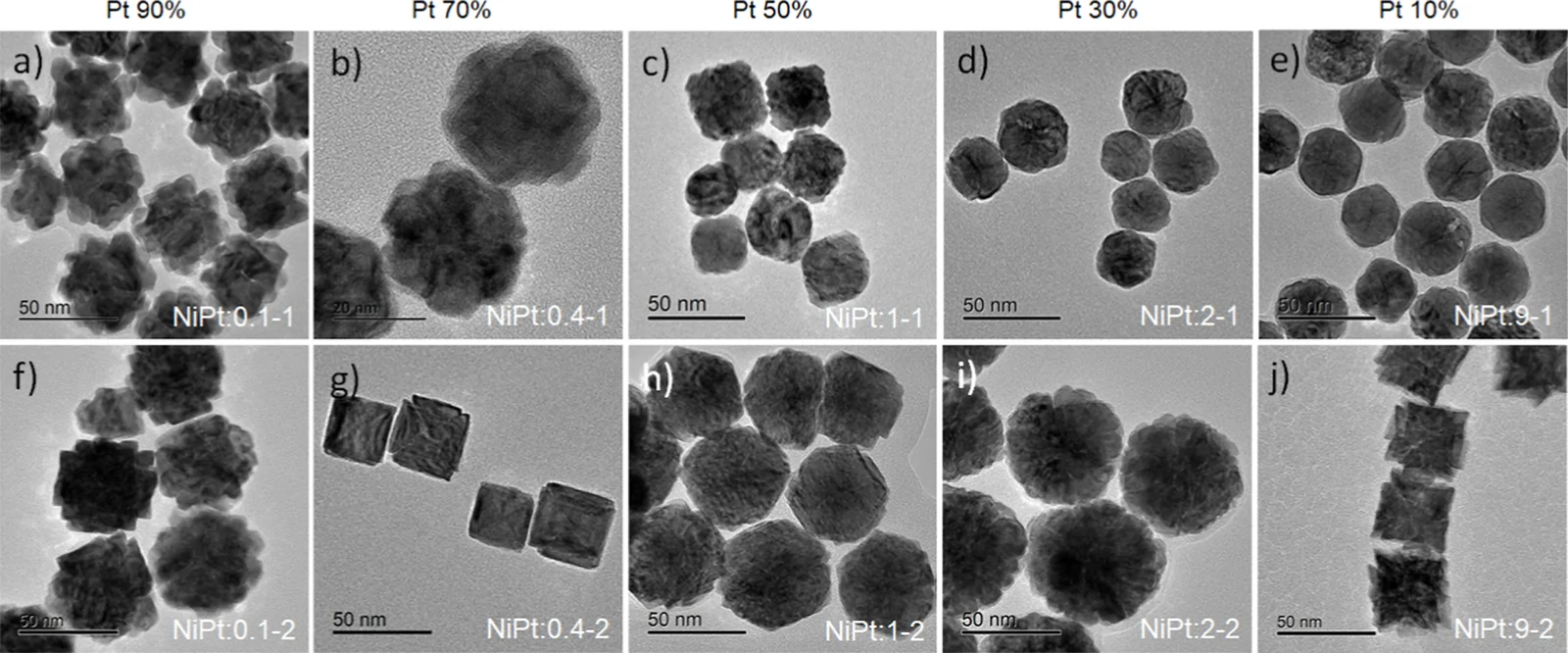
TEM images of the obtained Ni–Pt nanoparticles produced fixing the PDDA volume (1 mL, upper row, and 2 mL, bottom row) and varying the nominal metal concentration. The relative percentage of Pt­(acac)_2_ used for the synthesis is indicated. (a) NiPt:0.1-1, (b) NiPt:0.4-1, (c) NiPt:1-1, (d) NiPt:2-1, (e) NiPt:9-1, (f) NiPt:0.1-2, (g) NiPt:0.4-2, (h) NiPt:1-2, (i) NiPt:2-2, and (j) NiPt:9-2.

**Table 2:** Experimental Parameters and Chemical Composition Obtained by Changing the Relative Metal Precursor Concentrations and Keeping Constant the PDDA Used for the Synthesis of Ni–Pt Nanoparticles

sample ID	figure	PDDA (mL)	EG/PDDA	NiCl_2_ (μmol)	Pt_(acac)_ (μmol)	Ni/Pt ratio	Ni at%–Pt at% (EDS)
NiPt:0.1-1	[Fig fig3]a	1	20	2	18	0.11	9–91
NiPt:0.4-1	[Fig fig3]b	1	20	6	14	0.42	33.3–66.7
NiPt:1-1	[Fig fig1]c, 2b, 3c	1	20	10	10	1	50–50
NiPt:2-1	[Fig fig3]d	1	20	14	6	2.3	48–52
NiPt:9-1	[Fig fig3]e	1	20	18	2	9	64.2–35.8
NiPt:0.1-2	[Fig fig3]f	2	10	2	18	0.11	10–90
NiPt:0.4-2	[Fig fig3]g	2	10	6	14	0.42	42.7–57.3
NiPt:1-2	[Fig fig1]d, 2c, 3h	2	10	10	10	1	48–52
NiPt:2-2	[Fig fig3]i	2	10	14	6	2.3	62–38
NiPt:9-2	[Fig fig3]j	2	10	18	2	9	34–66

Interestingly, when adding 2 mL of PDDA, the particles underwent a different morphological evolution (Figure 3f–j). For Pt-rich compositions, stepped NPs were obtained (NiPt:0.1-2). However, as Ni content increased, the morphology transitioned abruptly from stepped NPs to stepped cubic-like NPs and subsequently to multibranched particles with sharp edges, as shown in Figure 3. At the Ni-rich edge (NiPt:9-2), the NPs displayed a complex branched cube-like shape with concave facets, suggesting a preferential growth at the edges and corners. The actual chemical compositions for the Pt-rich region in the 2 mL series had a good agreement with the nominal compositions. In contrast, for particles with 90–70% Ni range, the NPs exhibited a Ni deficit, reflecting the preferential reduction of Pt and the limited capacity of EG to completely reduce Ni^2+^ ions due to their difference in reduction potential between the metal precursors.

The crystalline structures of representative samples NiPt:0.1-1, NiPt-0.4-1, NiPt:0.1-2, and NiPt:9-2 were determined by HR-TEM and HAADF imaging (Figure 4) and SAED (Figure S8). The high-magnification images in Figure 4a,e show the highly branched morphology of the NiPt:0.1-1 NPs. By viewing a single particle along the [001] zone axis, highlighted by its fast Fourier transform (FFT) in the middle row, it can be observed this multibranched particle formed by the overgrowth of truncated octahedral branches on the corners and facets of a hypothetical central cube. In contrast, when synthesis proceeded under a Ni-rich environment (NiPt:9-1), no branched NPs were formed (Figure 4b,f). Nevertheless, a stepped surface remains visible in these high-magnification images, and some particles displayed twin boundaries which can alter their external shape. When the PDDA concentration doubled to 2 mL, more complex stepped branched NPs with sharp edges formed for the Pt-rich sample (NiPt:0.1-2), Figure 4c,g. Finally, for the NiPt:9-2 sample, concave stepped shapes were obtained with a single-crystalline structure, as shown in Figure 4d,h. The morphological changes are easily visible from the HAADF images, highlighting PDDA’s role in modulating surface energies during growth. In the HAADF images, the [001]_FCC_ direction is indicated to highlight the differences in the shape and crystal growth.

**Figure 4:**
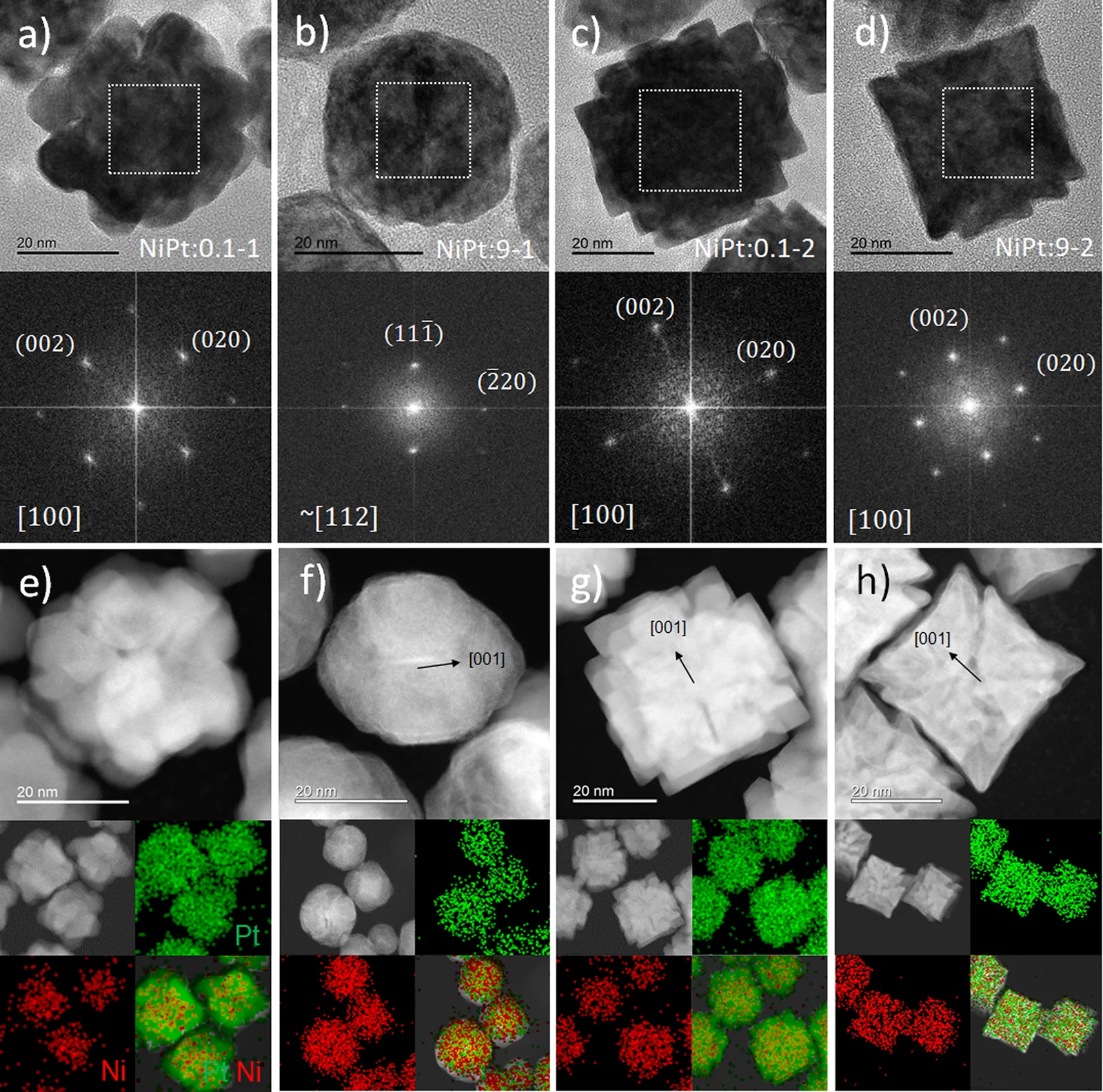
HR-TEM images and their FFT of the indicated areas (top), and HAADF-imaging and EDS mappings (bottom) of the Ni–Pt NPs produced fixing the PDDA volume to 1 mL or 2 mL, and varying the Ni/Pt ratio. (a,e) NiPt:0.1-1; (b,f) NiPt:9-1; (c,g) NiPt:0.1-2; and (d,h) NiPt:9-2.

### 3.3 Determination of Catalytic Performance in the 4-Nitrophenol Reduction

Shape variations produce different surface structures that can be applied for catalytic reactions since different crystallographic facets and atomic ordering are exposed at the surface. Six representative shapes were chosen to test their catalytic activity, namely NiPt:1-0.5, NiPt:1-1, NiPt:1-4, NiPt:0.1-1, NiPt:0.1-2, and NiPt:9-2, taking as the model reaction the 4-nitrophenol reduction in the presence of NaBH_4_, Figure 5. The catalytic test was performed in a 3 mL quartz cuvette, following the changes at 400 nm of the 4-nitrophenolate ion after injection of the catalysts. Samples were dried, weighed, and redispersed in water. Initially, different sample concentrations were tested to optimize the catalyst’s amount.

**Figure 5:**
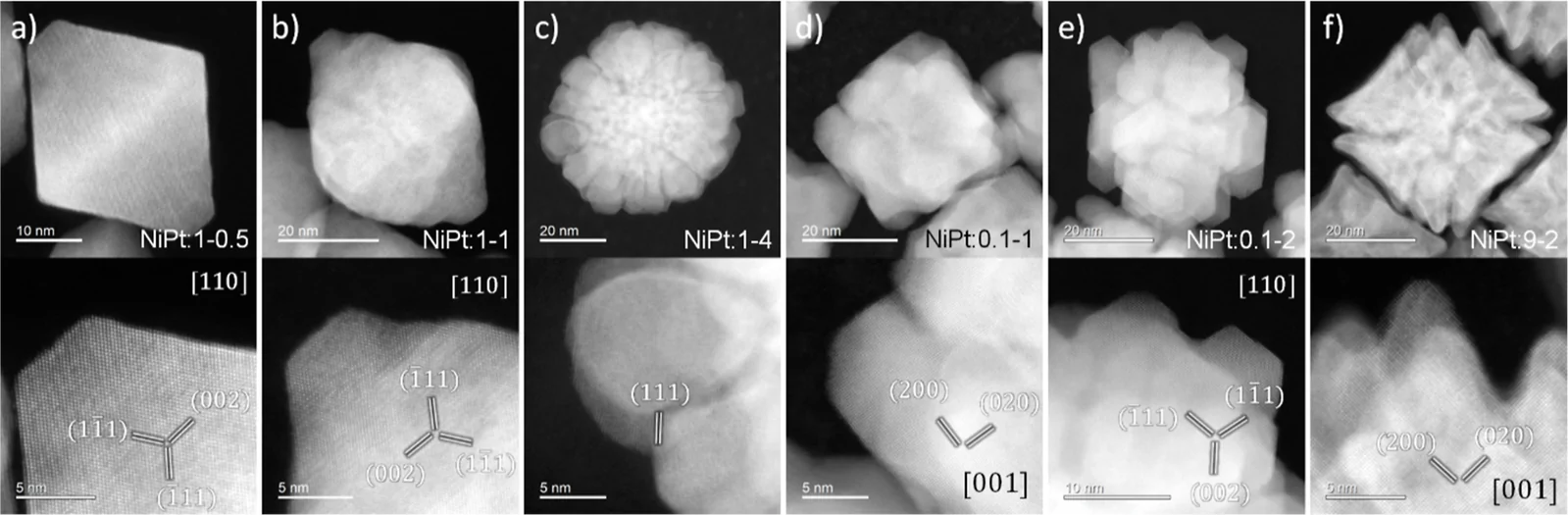
High-resolution HAADF images of the shape variations obtained by our PDDA-assisted polyol method and used as model catalysts for the 4-nitrophenol reduction. (a) NiPt:1-0.5, (b) NiPt:1-1, (c) NiPt:1-4, (d) NiPt:0.1-1, (e) NiPt:0.1-2, and (f) NiPt:9-2.

All the produced nanoalloys showed excellent catalytic activity toward the model reaction, converting more than 90% of the 4-NP to 4-AP in the first 10 min of reaction with catalyst load less than 30 μg. Plots of In­(*A*/*A*_0_) versus time are presented as insets in Figure 6 for samples NiPt:1-0.5, NiPt:1-1, NiPt1-4, NiPt:0.1-1, NiPt:0.1-2, and NiPt:9-2 as a function of catalyst loading. The catalytic activities were compared to that of synthesized Pt NPs, Ni NPs, and commercial Pt and Pt/C nanoparticles as reference samples, Figure S9. The synthesized Pt NPs showed a faster reduction for the 4-NP reduction compared to the NiPt and commercial NPs. The first-order apparent rate constant (*k*_app_) was calculated from these curves by treating them as pseudo-first-order reactions. The calculated *k*_app_ for each sample was plotted for a better visualization of the trends and added as insets.

**Figure 6:**
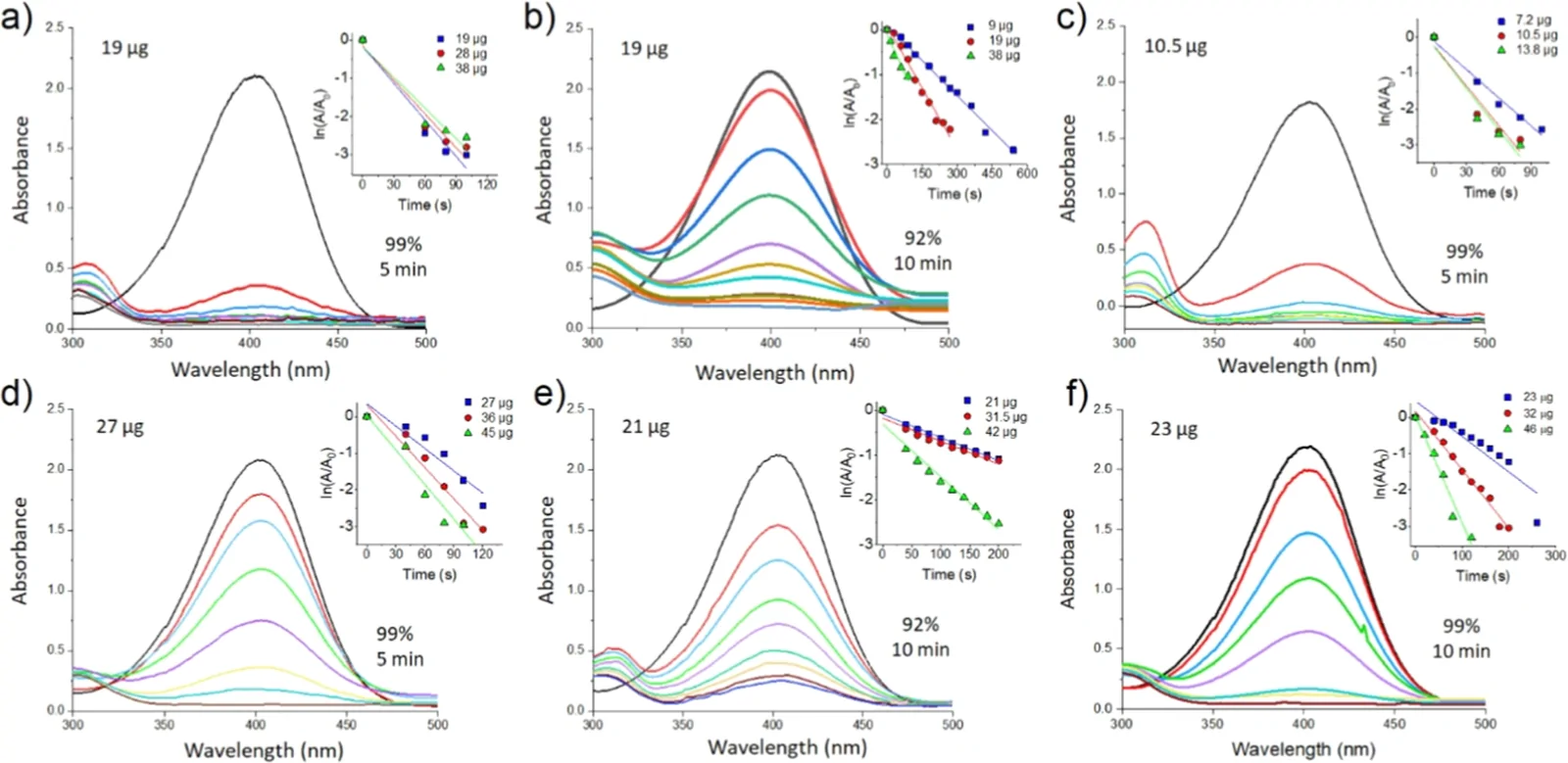
UV–vis monitoring of the 4-NP reduction to 4-AP as a function of time for the different catalysts. Absorbance curves correspond to the indicated metal load. Insets present the ln­(*A*/*A*_0_) graphs for reaction constant calculations of the different samples as a function of the catalyst loading: (a) NiPt:1-0.5, (b) NiPt:1-1, (c) NiPt:1-4, (d) NiPt:0.1-1, (e) NiPt:0.1-2, and (f) NiPt:9-2.

The higher values of the apparent rate for the selected samples increased as follows: 0.72 min^–1^ (NiPt:0.1-2) < 1.77 min^–1^ (NiPt:9-2) < 1.373 min^–1^ (Pt) < 1.506 min^–1^ (NiPt:1-1) < 1.884 min^–1^ (NiPt:0.1-1) < 2.232 min^–1^ (NiPt:1-0.5) < 3.084 min^–1^ (NiPt:1-4). For a very low catalyst load range (<30 μg), samples NiPt:1-4, NiPt:1-0.5, NiPt:1-1, and NiPt:0.1-1 presented the higher values. Increasing the catalyst load (>30 μg), samples NiPt:1-1, NiPt:0.1-1, NiPt:0.1-2, and Pt NPs enhanced their activity. Although the *k*_app_ values are of great relevance for comparison with other systems, the mass constants (*k*_m_) have a greater impact since they consider the amount of catalyst used in a given reaction. Table 3 shows the corresponding data for the 4-nitrophenol reduction including the values of *k*_m_. The best obtained *k*_m_ values corresponded to the samples NiPt:1-4 (3738.2 s^–1^ g^–1^), NiPt:1-0.5 (1957.9 s^–1^ g^–1^), NiPt:1-1 (857.7 s^–1^ g^–1^), and Pt NPs (1069.9 s^–1^ g^–1^). More importantly, the alloy structure of the herein reported Ni–Pt NPs reduced 50% of the Pt use, increasing their activity with a high *k*_app_ and *k*_m_ values compared to Pt NPs.

**Table 3:** Calculated *K*_app_ and *k*_m_ Values for Each Sample as a Function of the Catalyst Loading

sample ID	Pt % (EDS)	Pt % (ICP–MS)	mass (μg)	time (s)	*k*_app_ (s^–1^)	*k*_app_ (min^–1^)	*k*_m_ (s^–1^ g^–1^)
NiPt:1-0.5	52.1	49.0	19	80	0.0372	2.232	1957.9
			28	80	0.0336	2.016	1200
			38	80	0.0307	1.842	807.9
NiPt:1-1	50	48.8	26	80	0.0223	1.338	857.7
			39	80	0.0233	1.398	597.4
			45.5	80	0.0251	1.506	551.6
NiPt:1-4	66.7	60.8	7.15	100	0.0211	1.266	2951.1
			10.45	100	0.0321	1.926	3071.8
			13.75	100	0.0514	3.084	3738.2
NiPt:0.1-1	91	-	27	120	0.0200	1.200	740.7
			36	120	0.0285	1.710	791.7
			45	120	0.0314	1.884	697.8
NiPt:0.1-2	90	-	21	200	0.0067	0.402	319
			31.5	200	0.0085	0.510	269.8
			42	200	0.0120	0.720	286
NiPt:9-2	66	-	23.0	200	0.0098	0.598	426.1
			32.2	200	0.0161	0.966	500.0
			46.0	200	0.0295	1.770	641.3
Pt-NPs	100	-	14.3	240	0.0153	0.918	1069.9
			28.5	240	0.014	0.840	491.2
			57	240	0.0229	1.373	401.8
Ni-NPs	0	-	95	1800	0.0019	0.114	19.8

It has been shown in previous studies that the catalytic activity of nanoparticles is favored when they have protuberances, pores, high Miller index surfaces, defects, etc., since these promote the interaction between the reagents and the catalyst by accelerating their adsorption, in addition to having a greater number of active sites.^4,22^ We observed that the most catalytically active samples corresponded to NiPt:1-1, NiPt:1-0.5, and NiPt:1-4. The elemental composition estimation was complemented by inductively coupled plasma mass spectrometry (ICP–MS) for the most catalytically active samples. Hence, high-magnification HAADF imaging was performed on these samples aiming to provide a description of the different environments where the catalytic reaction occurred and whose application can be extended to other reactions, Figure 7.

**Figure 7:**
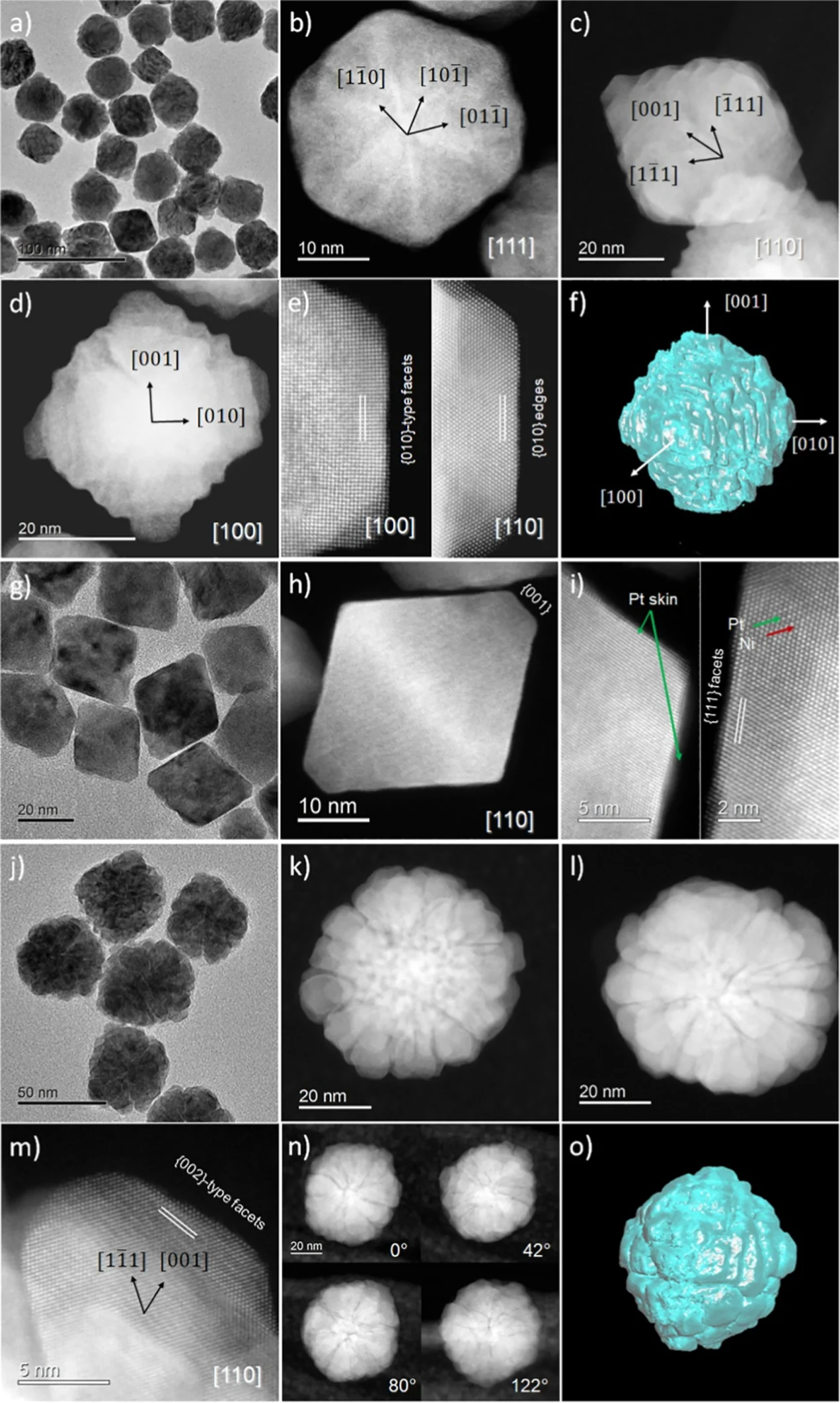
HAADF imaging of selected nanoalloys showing the different crystal growth and faceting: (a–f) NiPt:1-1; (g–i) NiPt:1-0.5; and (j–o) NiPt:1-4.

From Figure 7a, the NiPt:1-1 sample showed good shape homogeneity, where the differences proceed from the projections of the complex 3D shape of the particles viewed at different orientations. The high-magnification images in Figure 7b,c correspond to a particle viewed from the [111], [110], and [100] directions, showing the different projected external shape. Based on the growth directions and the observed mass–thickness contrast, the shape was determined as the pyramidal morphology well described by Bazán-Díaz,^7^ in which particles presented stepped overgrowth along ⟨100⟩ directions, with nickel and platinum well dispersed into a face-centered cubic solid solution in a proportion close to 50-50, substantially reducing the Pt consumption for the reaction. A detailed observation of the particle edges, when viewing from ⟨100⟩ and ⟨110⟩ projections, revealed the features of the {010}-terminated facets and high-index edges, Figure 7e,f. No segregation was observed confirming the solid solution mixing of the metals. Moreover, a tomographic reconstruction was performed for a better visualization of the stepped 3D shape.

The NiPt:1-0.5 sample presented a more regular shape resembling a truncated octahedron, exposing extended {111} facets and small {100} facets, Figure 7g. The average Pt content slightly increased to 52.1%, indicating that at lower PDDA concentrations, fewer nickel ions were reduced and reflecting the soft-reducer role of the electrolyte. Interestingly, high-magnification images revealed the structures of these particles. The particles presented atomic segregation, forming a Pt-skin composed by one to three atomic surface layers revealed by the higher contrast of these layers, Figure 7h,i. It has been reported that {111} facets and Pt-skin configurations enhance the catalytic activity of Ni–Pt nanoparticles toward different catalytic reactions. This is due to the exposure of Pt on the surface and a strain effect. It is seen that the activity was similar to that of the reference Pt nanoparticles, which presented a truncated octahedron morphology. However, the atomic composition reduced the amount of Pt used for the reaction by half. Moreover, it is noted that at the subsurface and particle core a partial ordering of the Ni–Pt alloy took place.

In turn, NiPt:1-4 presented a polycrystalline multibranched structure, formed by the apparent agglomerated small particles around a core, Figure 7j–o. The high-magnification images of one branch show that Ni and Pt are well distributed into its structure, forming a complete solid solution, with no observable surface segregation. A particle was imaged over a range of tilting angles for a better visualization of its 3D morphology, Figure 7n, and a tomographic reconstruction was performed, Figure 7o. This complex multibranched structure forms channels and a rough surface causing high activity toward the model reaction.

Finally, the catalytic activity parameters of the herein described nanoalloys were compared with other similar alloys available elsewhere, and a complete comparative table is presented in Table S3. Supported and unsupported catalysts were considered in the 4-NP reduction. A comparison between apparent and mass constants of each system of this work and other reported particles is presented, demonstrating that our particles presented a high activity in this model reaction.

## 4 Conclusions

In summary, we presented a protocol for shape tailoring of non-conventional Ni–Pt nanoparticles, using the PDDA polymer as a shape modulator agent. The simple variation of the PDDA concentration and Ni/Pt proportion during crystal growth induced important morphological and structural changes, achieving complex shapes varying from intricate concave particles to stepped structures and multibranched nanoparticles. The catalytic activity of the different nanoalloys was compared for the 4-nitrophenol reduction, a model reaction of environmental relevance, obtaining high activity with very low catalyst loading. The use of these nanoparticles can be translated to other important reactions where the complex morphology and channel structure can be exploited. The herein reported synthesis represents a simple methodology to obtain water-soluble complex Ni–Pt nanostructures which present different surface environments, reducing the Pt consumption and catalyst loading.

## Supplementary Material



## Notes

Sketch of synthesis and purification protocol of Ni–Pt nanoalloys, additional TEM images and particle size distributions; SAED analysis; tables of chemical compositions determined by EDS and SAED analyses, and comparative table of Ni, Pt, and Pt NPs reported elsewhere for the nitrophenol reduction (PDF)
