# Supplementary material for: Shape Tailoring of Nonconventional Ni–Pt Nanoparticles through a PDDA-Polyol Method
Source: ACS Omega. 2026 Sep 9;11(37):55251–60. doi: 10.1021/acsomega.6c03056 (PMC13618817; doi:10.1021/acsomega.6c03056)
Supplement: Supplementary file 1 [file ao6c03056_si_001.pdf]

Supporting Information

Shape Tailoring of Non-conventional Ni – Pt Nanoparticles Through a PDDA-Polyol Method

Brayan Maqueda, Ariadna Pérez-Pérez, Alexis Hellmer, Miriam M. Tellez Cruz, Lourdes Bazán-Díaz,* and Rubén Mendoza-Cruz.**

Instituto de Investigaciones en Materiales, Universidad Nacional Autónoma de México. Circuito Exterior, Ciudad Universitaria, Ciudad de México, 04510. México.

Corresponding authors:

* bazanlulu@materiales.unam.mx

** rmendoza@materiales.unam.mx

CONTENT:

Chemical composition determination from electron diffraction

Figure S1. Sketch of the synthesis and purification protocol of Ni-Pt nanoalloys in solution.

Figure S2. Photograph of the final Ni-Pt samples as a function of the PDDA content.

Figure S3. Low magnification TEM images of the NiPt NPs produced by changing the PDDA volume.

Figure S4. Size histograms for samples in Figure S3.

Figure S5. Low magnification TEM images and SAED patterns of the NiPt NPs produced by changing the PDDA volume while keeping the Pt/Ni ratio constant.

Table S1. Chemical compositions of representative samples determined by EDS and electron diffraction analysis.

Figure S6. Low magnification TEM images of the NiPt NPs produced by varying relative ratios between metal precursors and PDDA.

Figure S7. Size histograms for samples in Figure S6.

Figure S8. Low magnification TEM images and SAED patterns of the NiPt NPs produced by changing the PDDA volume and Pt/Ni ratio.

Table S2. Chemical compositions of representative samples determined by EDS and electron diffraction analysis.

Figure S9. UV-Vis monitoring of the reduction of 4-nitrophenol to using Pt and Ni nanoparticles as control catalyst.

Table S3. Comparative table of Ni, Pt, and Ni-Pt nanoparticles reported elsewhere for the nitrophenol reduction.

Chemical composition determination from electron diffraction

The chemical composition of the alloy was calculated through electron diffraction pattern analysis and compared with EDS results. The estimation was performed using Vegard's Law,[25, 26] relating the crystal lattice parameters to the composition of each element.

$$a_{alloy} = a_A(1 - x) + x a_B$$

Where a_{alloy}, a_A, and a_B are the lattice parameters of the alloy, element A, and element B, respectively, and x is the composition of element B.

The interplanar spacing values were taken into account, so the equation can be rewritten as follows:

$$x_B = \frac{d - d_A}{d_B - d_A}$$

Where x is the composition of element B and d is the interplanar spacing of the alloy (d), element A (d_A), and element B (d_B). Pure Pt and Ni nanoparticles were used as reference for lattice distances measurements.

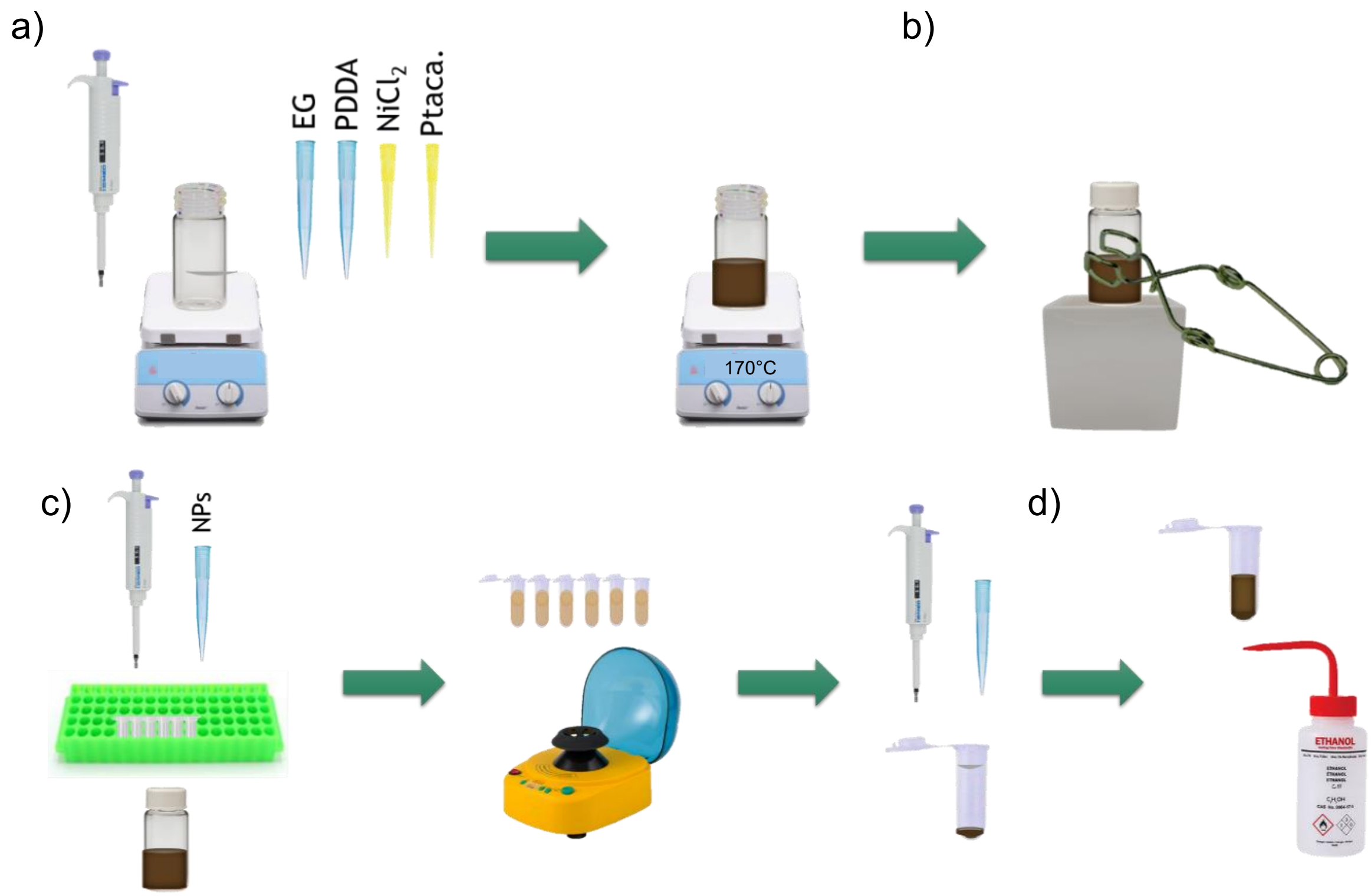

Figure S1. Sketch of the synthesis and purification protocol of Ni-Pt nanoalloys in solution. (a) Mixture of reactants, (b) Heating to the desired temperature for 1 h and remotion from the heat source. (c) Purification by washing with ethanol / acetone and centrifugation. (d) Redispersion in water.

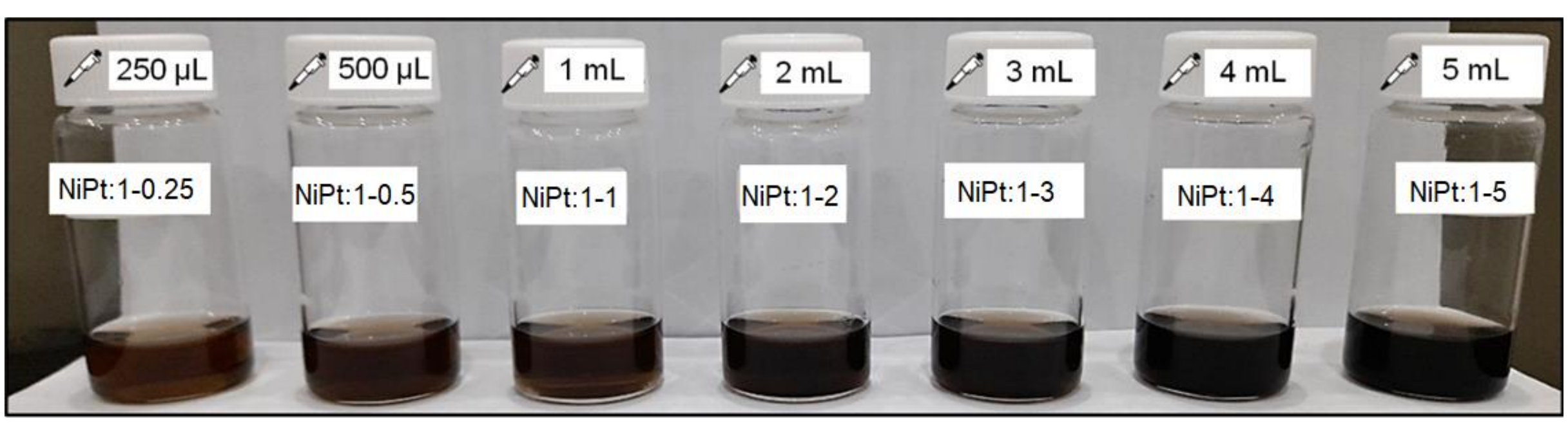

Figure S2. Photograph of the final Ni-Pt samples as a function of the PDDA content (representative volumes): 250 µL (NiPt:1-0.25), 500 µL (NiPt:1-0.5), 1 mL (NiPt:1-1), 2 mL (NiPt:1-2), 3 mL (NiPt:1-3), 4 mL (NiPt:1-4), and 5 mL (NiPt:1-5).

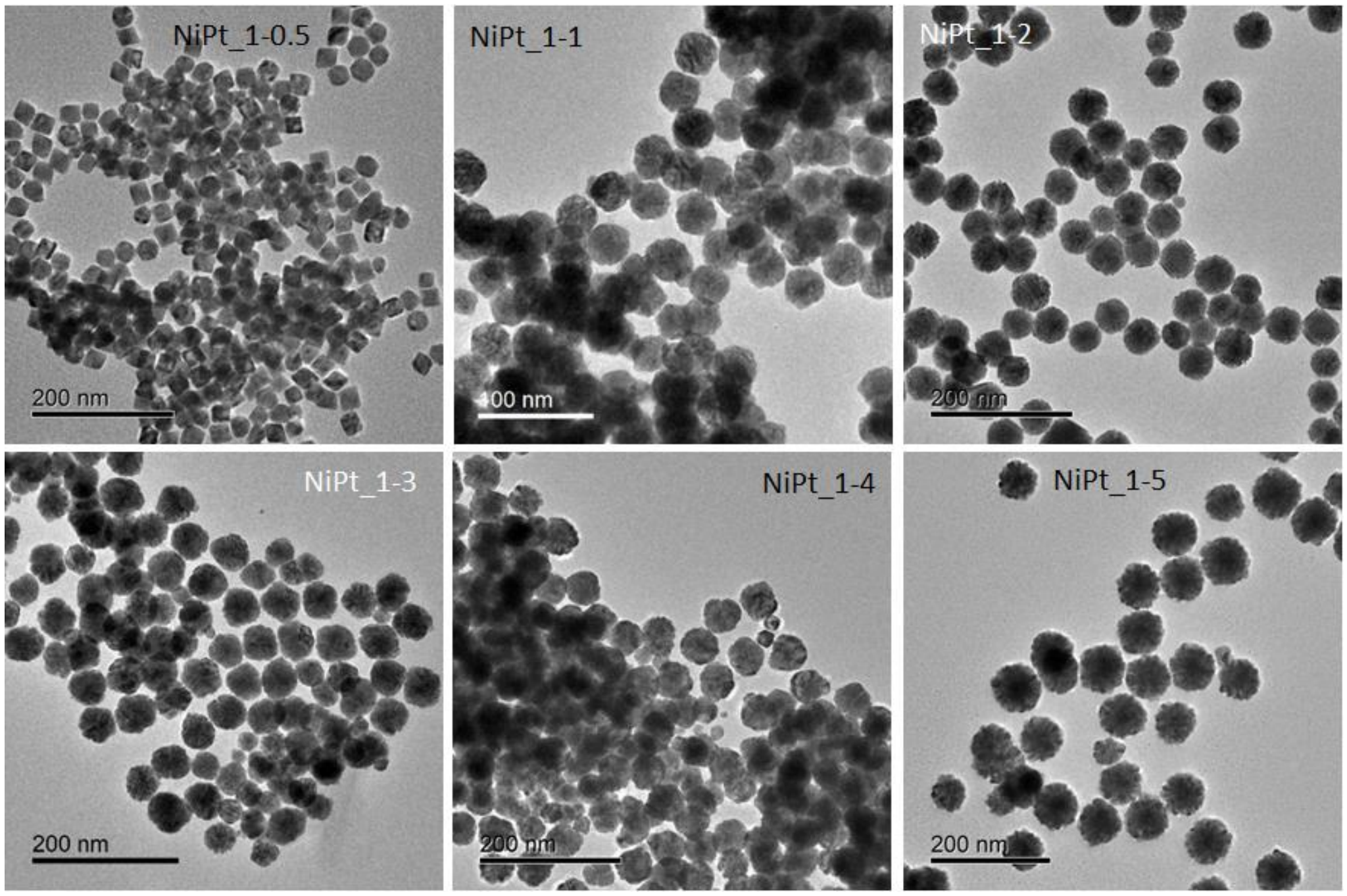

Figure S3. Low magnification TEM images of the NiPt NPs produced by changing the PDDA volume while keeping the Pt/Ni ratio constant. Nomenclature: First number indicating the Ni/Pt ratio; second number the PDDA volume in ml.

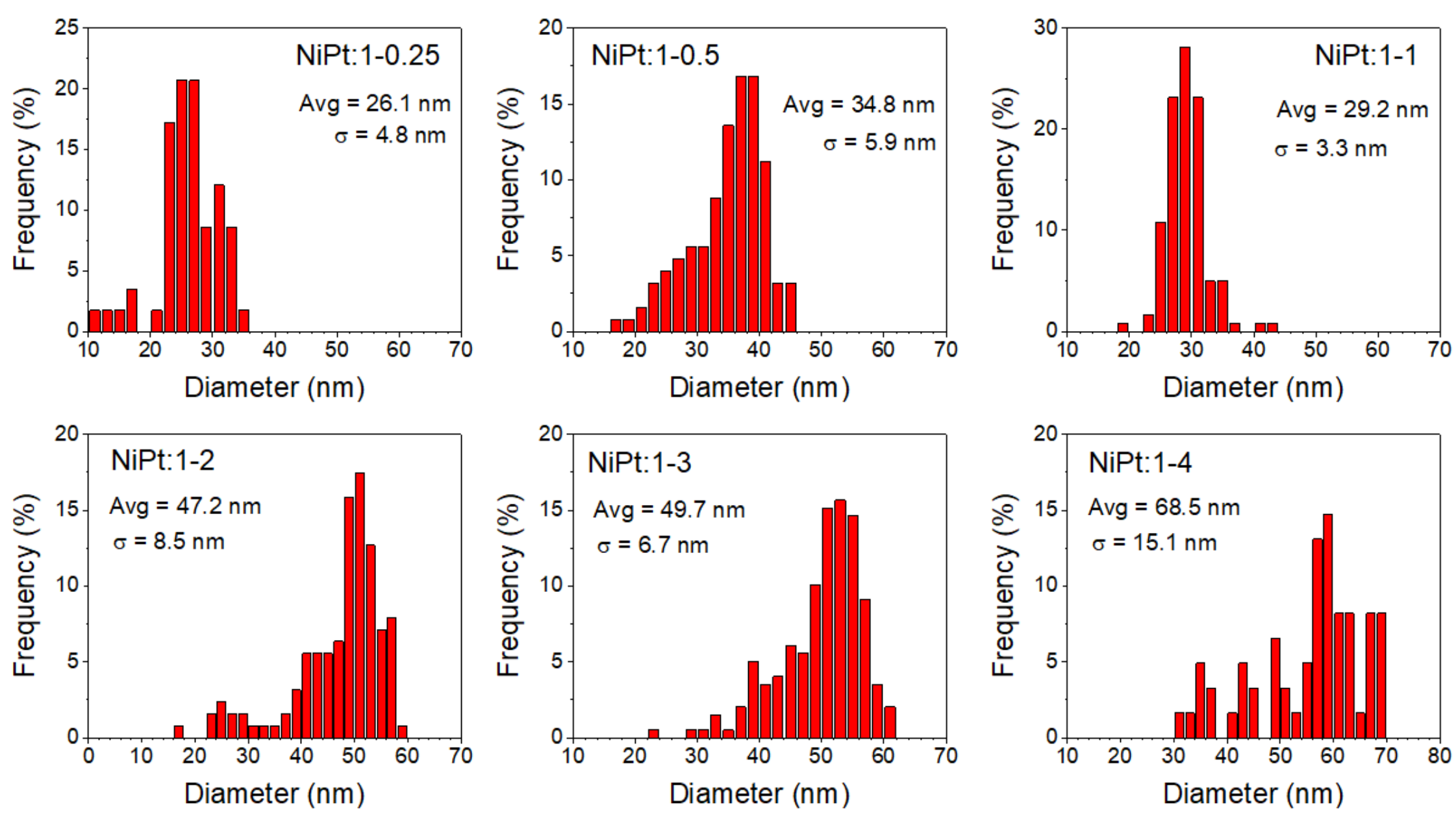

Figure S4. Size histograms for samples in Figure S3, where PDDA volume was varied while keeping the Pt/Ni ratio constant.

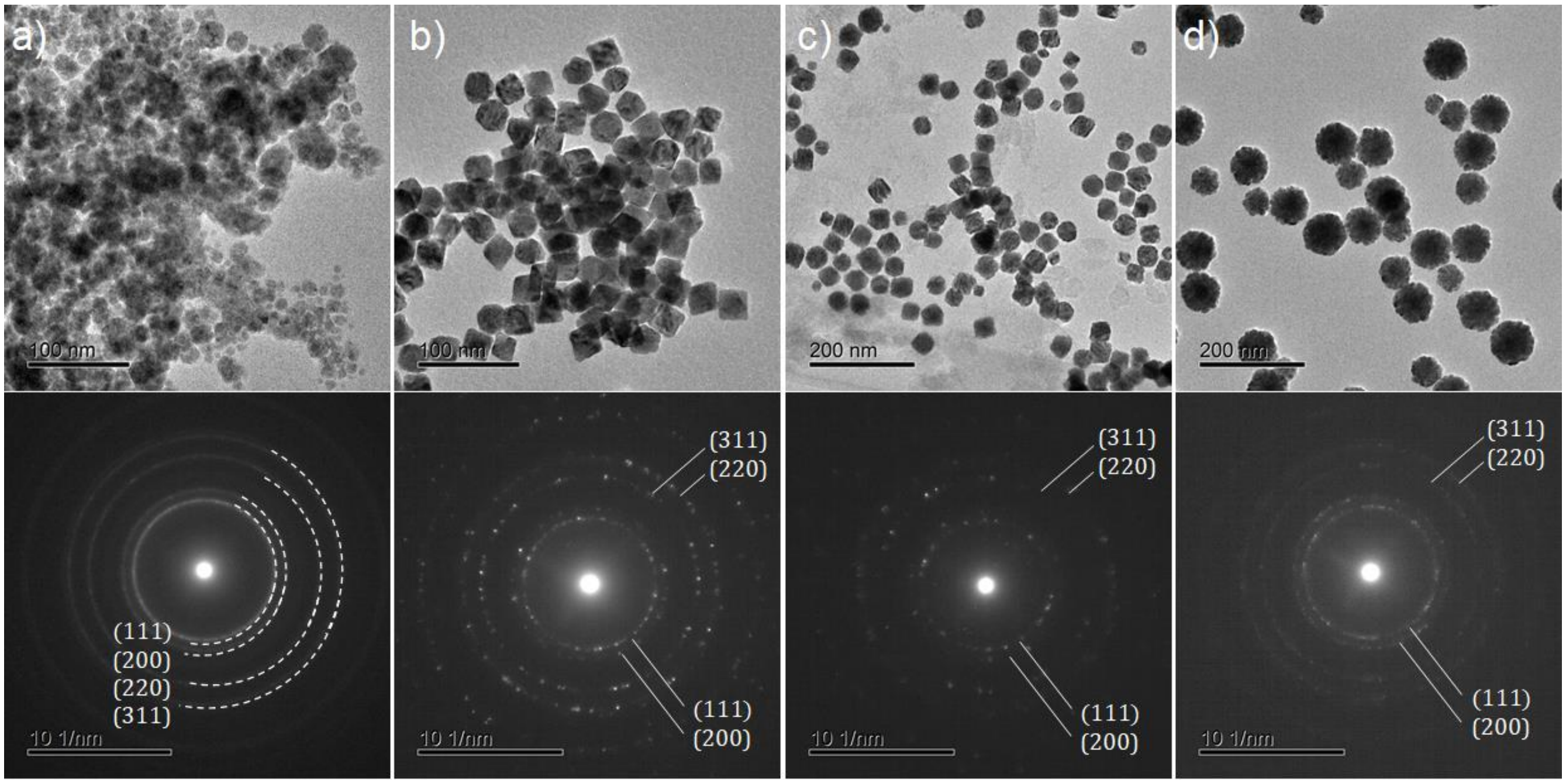

Figure S5. Low magnification TEM images and SAED patterns of the NiPt NPs produced by changing the PDDA volume while keeping the Pt/Ni ratio constant. Pure nickel NPs were synthesized as a control sample. a) Ni NPs, b) NiPt:1-0.5, c) NiPt:1-1, d) NiPt:1-4. The patterns display lattice reflections corresponding to an FCC structure.

Table S1. Chemical compositions of representative samples determined by EDS and electron diffraction analysis.

**Sample ID**	**Figure**	**PDDA (ml)**	**$NiCl_2$ (µmol)**	**$Pt_{(acac)}$ (µmol)**	**Ni % - Pt % (EDS)**	**Ni % - Pt % (SAED)**	**SAED cell parameter (Å)**
Pt				20	0 – 100		3.94
NiPt:1-0.5	1b, 2a	0.5	10	10	47.9 – 52.1	45 – 55	3.76
NiPt:1-1	1c, 2b	1	10	10	49.3 – 50.7	49 – 51	3.74
NiPt:1-2	1d, 2c	2	10	10	48 – 52	48 – 52	3.75
NiPt:1-4	1f, 2d	4	10	10	33.3 – 66.7	35 – 65	3.67
Ni			20		100 – 0		3.53

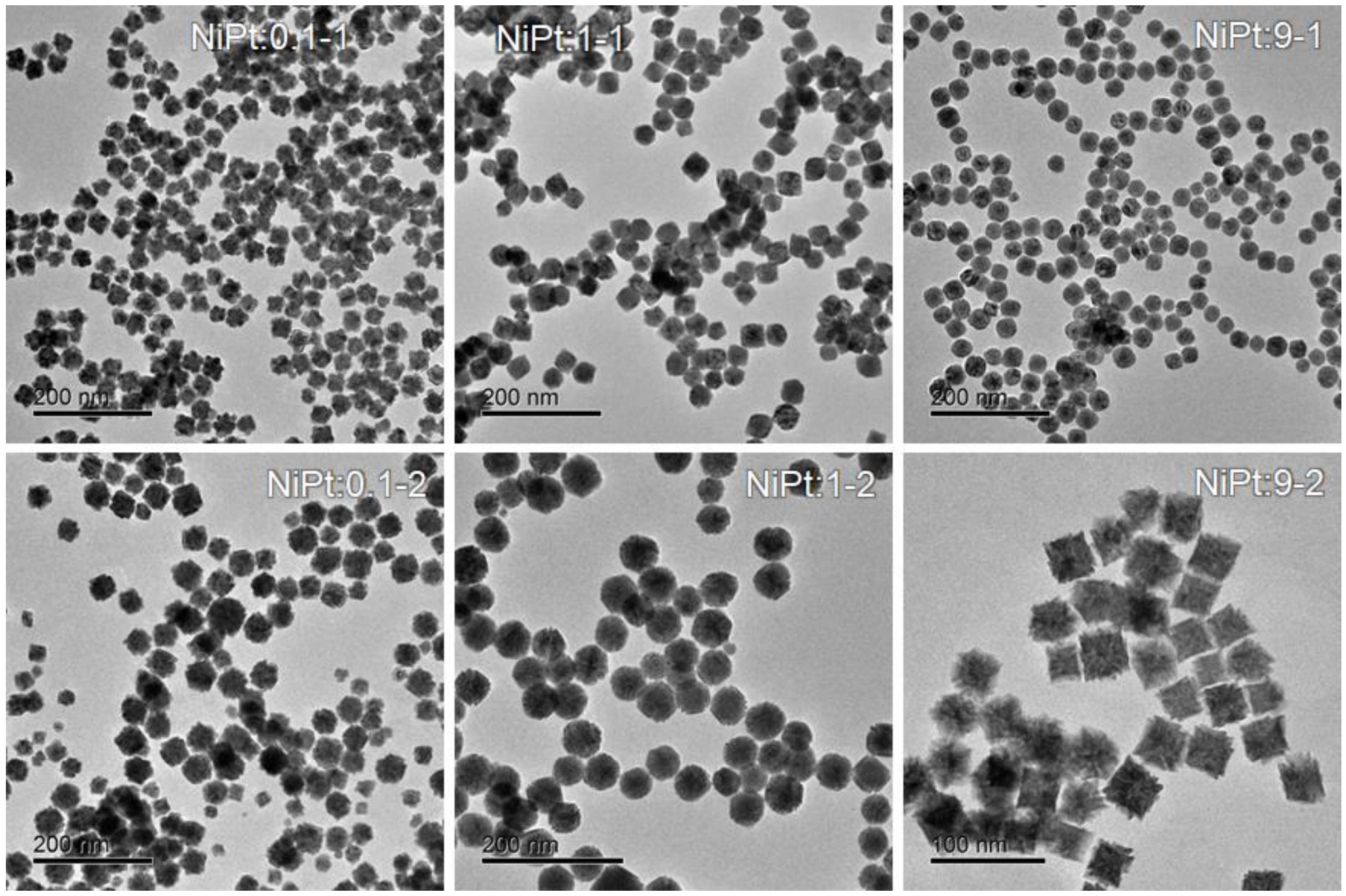

Figure S6. Low magnification TEM images of the NiPt NPs produced by varying relative ratios between metal precursors and PDDA. Nomenclature: First number indicating the Ni/Pt ratio; second number the PDDA volume in ml.

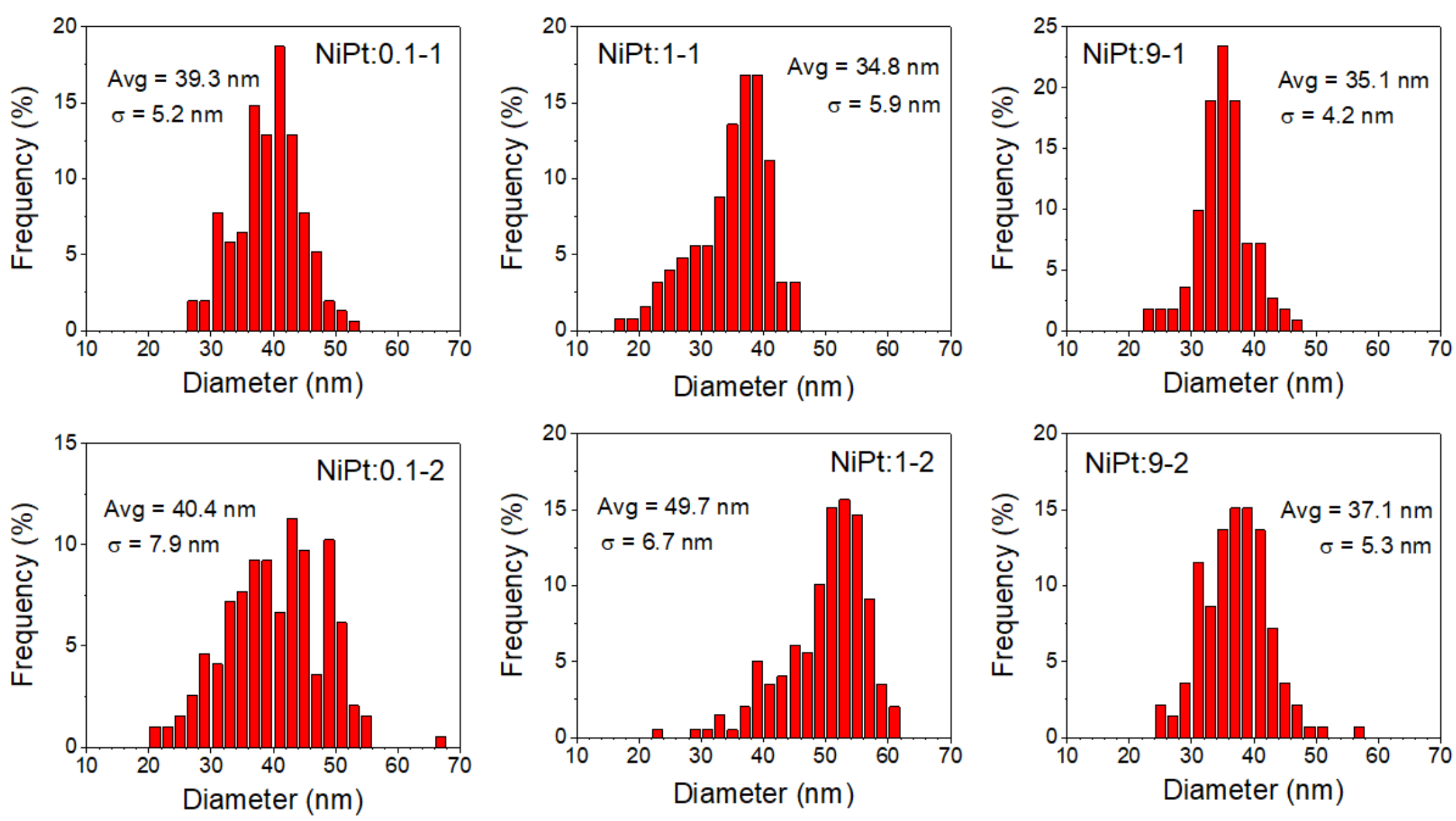

Figure S7. Size histograms for samples in Figure S6, where relative ratios between metal precursors and PDDA were varied.

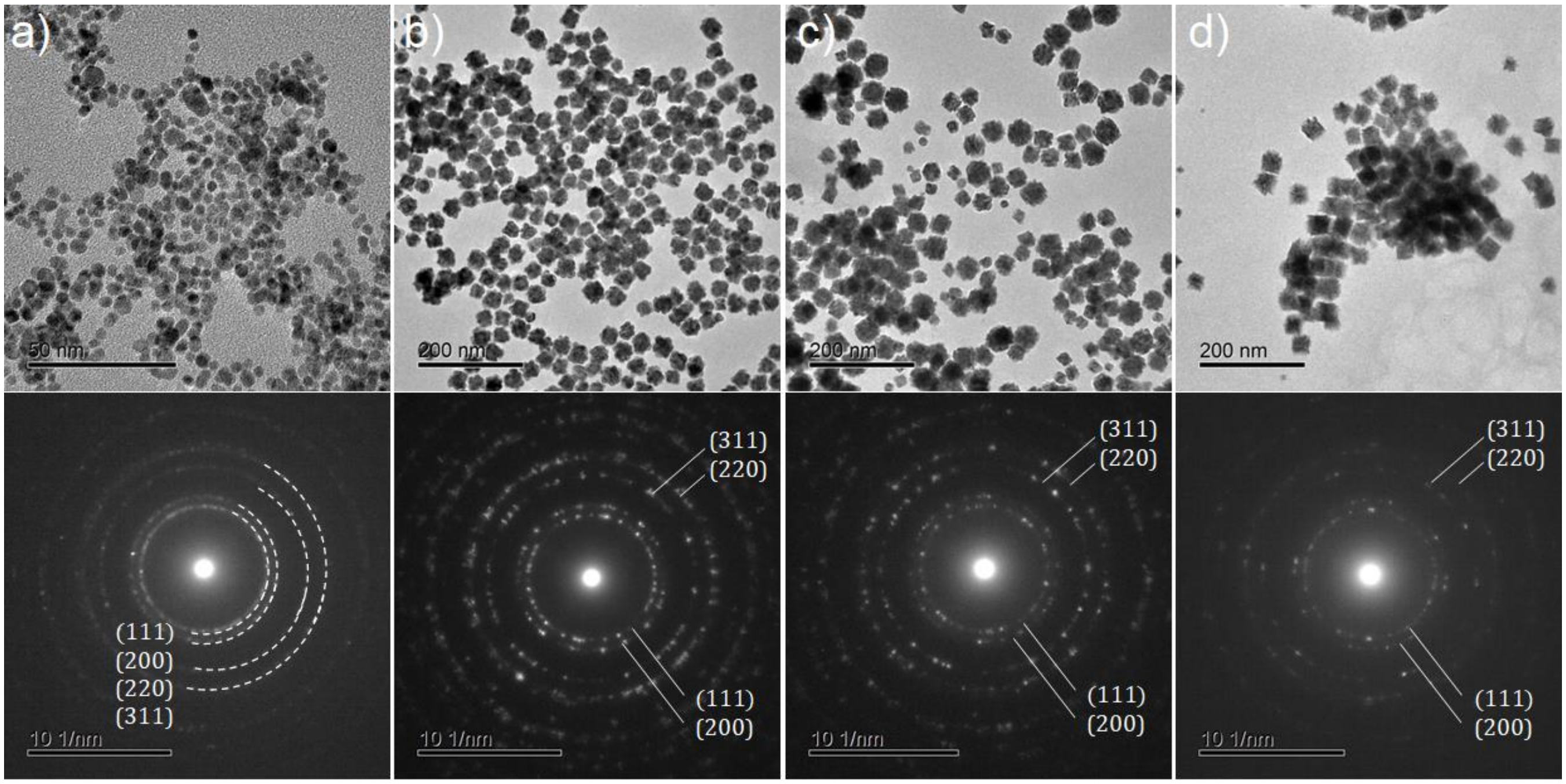

Figure S8. Low magnification TEM images and SAED patterns of the NiPt NPs produced by changing the PDDA volume and Pt/Ni ratio. Pure Pt NPs were synthesized as a control sample for the determination of lattice parameter and catalytic activity. a) Pt NPs, b) NiPt:0.1-1, c) NiPt:0.1-2, d) NiPt:9-2. The patterns display lattice reflections corresponding to an FCC structure.

Table S2. Chemical compositions of representative samples determined by EDS and electron diffraction analysis.

Sample ID	Figure	PDDA (ml)	$NiCl_2$ (µmol)	$Pt_{(acac)}$ (µmol)	Ni % - Pt % (EDS)	Ni % - Pt % (SAED)	SAED cell parameter (Å)
Pt			-	20	0 – 100		3.94
NiPt:0.1-1	3a, 4a	1	2	18	9 – 91	10 – 90	3.90
NiPt:1-1	1c, 3c	1	10	10	49.3 – 50.7	49 – 51	3.74
NiPt:9-1	3e, 4b	1	18	2	64.2 – 35.8	65 – 35	3.67
NiPt:0.1-2	3f, 4c	2	2	18	10 – 90	9 – 91	3.90
NiPt:1-2	1d, 3h,	2	10	10	48 – 52	48 – 52	3.75
NiPt:9-2	3j, 4d	2	18	2	34 – 66	45 – 55	3.76
Ni			20	-	100 – 0		3.53

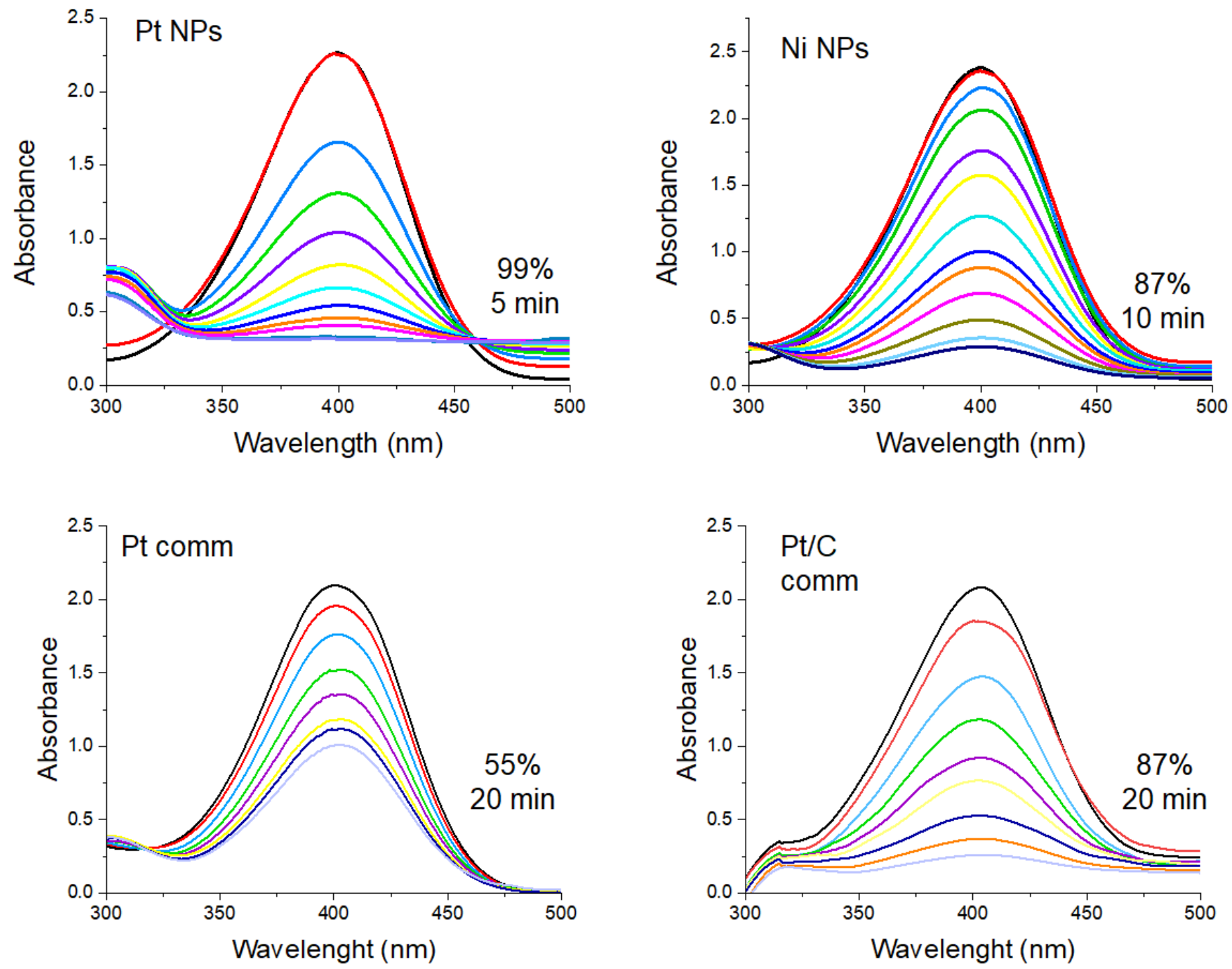

Figure S9. UV-Vis monitoring of the reduction of 4-nitrophenol using pure Pt and Ni NPs as control catalysts, as well as commercial Pt and Pt/C nanoparticles.

Table S3. Comparative table of Ni, Pt, and Ni-Pt nanoparticles reported elsewhere for the nitrophenol reduction.

#	Sample	Shape	Size (nm)	Catalyst mass (µg)	K_{app} [s^{-1}]	K_m [s^1g^{-1}]	Reaction time [s]	Maximum conversión	Ref.
**This Work**									
1	Ni_4Pt_5-0.5	Truncated octahedron	25	19	0.0372	1957.9	80	≈ 100.00 %	TW
2	NiPt-1	Stepped pyramidal	47	26	0.0223	857.7	80	> 92%	TW
3	Ni_1Pt_2-4	Branched	74	7.2	0.0211	2951.1	300	≈ 100.00 %	TW
4	Ni_1Pt_9-1	Protuberant	37	27	0.0200	740.7	300	≈ 100.00 %	TW
5	Ni_1Pt_9-2	Protuberant		21	0.0067	319.0	600	> 92%	TW
6	Ni_7Pt_3-2	Concave cubic		23	0.0098	426.1	600	> 99%	TW
**Unsupported Ni NPs**									
7	Ni NPs	Esférica	9.9 ± 4.6	95	0.0019	19.8	1800	87.8 %	27
8	Ni-C NPs	Nanobar	NR*	1000	0.0174	17.41	60	93.00 %	28
9	CTAB Ni NPs	Spherical - Irregular	150.0	3000	0.0010	0.33	960	NR*	29
10	PEG Ni NPs	Spherical	125.0	3000	0.0013	0.43	960	NR*	29
11	Gelatin Ni NPs	Snowflake	100.0	3000	0.0018	0.60	960	NR*	29
12	CTAB + PEG Ni NPs	Spherical	75.0	3000	0.0027	0.90	960	NR*	29
13	CTAB + Gelatin Ni NPs	Nanobar	20.0	3000	0.0024	0.80	960	NR*	29
14	Ni-SiO_2	Spherical	50.0	1000	0.0011	1.133	960	NR*	30
15	Ni NPs	Spherical	193.0	1800	0.0014	0.78	180	97 %	31
16	Ni NPs	Spherical	8.0	500	0.0007	1.40	480	53.80 %	32
17	Ni NPs	Spherical	60.0 ± 9.3	90000	0.0001	0.002	7200	80.10 %	33
**Supported Ni NPs**									
18	Ni/SNT	Nanotubular	≈ 6.5	920	0.0840	91.30	60	≈ 100 %	34
19	Ni/p (AMPS)	Spherical	50.0-100.0	5920	0.0009	0.16	3600	100 %	35
20	RGO/Ni	Spherical	2.0-4.0	6500	0.0002	0.04	8400	NR*	36
21	Ni-AG-sponge	Irregular-porosa	< 100.0	638	0.0026	4.12	480	95.00 %	37
22	Ni/ZnO	Nanowire	57.0	1000	0.0017	1.68	1800	> 90.00 %	38
23	DE/Ni/N-C-800	Amorphous	20.3	654.6	0.0350	53.47	90	100 %	39
24	Ni/NCMTs	Tubular	≈ 20.0	41	0.0057	139.0	480	≈ 100%	40
25	Ni/SiO_2-C	Spherical	10.0	140	0.0052	37.1	600	≈ 100 %	41
26	Ni/MnO-Al_2O_3-C	Flake	≈ 7.0	39.4	0.0054	13.7	600	100 %	42
27	Ni/C	Spherical	≈ 10.0	249	0.0075	30.12	360	≈ 100 %	43
**Unsupported Pt NPs**									
28	SA-Pt NPs	Cubic / Spherical	2.0 ± 0.1	80	0.0600	750.00	240	100 %	44
29	C-Pt NPs	Spherical	6.0 ± 1.0	71	0.0005	7.00	3600	> 90 %	45
30	P-Pt NPs	Spherical	6.0 ± 1.0	286	0.0300	105.00	60	> 90 %	45
31	Pt NPs	Flower	21.0	100	0.0007	7.00	≈ 6000	100.00 %	46
32	GG-s-Pt NPs	Spherical	6.2	48	0.0083	170.18	240	97 %	47
33	Pt NPs	Spherical	5.0	5000	0.0021	0.41	960	NR*	48
34	Pt NPs	Nanotube	≈ 49.0	15	0.0033	222.22	600	≈ 99 %	49
35	Pt NPs	Nanocluster	< 10.0	1000	0.0004	0.41	2700	NR*	50
36	Pt NPs	NR*	5.2	50000	0.0350	0.70	180	100 %	51
37	Pt NPs	NR*	20 ± 2.5	4	0.0001	31.25	1800	< 20 %	52

**Supported Pt NPs**									
38	meso-CeO_2/ Pt NPs	Spherical	4.0	300	0.0060	20.00	480	> 90 %	[53]
39	nano-CeO_2/ Pt NPs	Flake	10.0	300	0.0041	13.67	720	> 90 %	[53]
40	Pt/Fe_2O_3	Flower	1.5	5000	0.0040	0.80	720	100 %	[54]
41	Pt/BBSiO_x	Spherical	≈ 2.25	240	0.0120	50.00	180	100 %	[55]
42	Pt/EMSiO_x	Spherical	≈ 2.25	240	0.0240	100.00	80	100 %	[55]
43	Pt/HRSiO_x	Spherical	≈ 2.25	240	0.0290	120.00	105	100 %	[55]
44	Pt/SnO_2	Nanocluster	< 1.5	53.32	0.0297	557.00	120	100 %	[56]
45	Fe_3O_4-PDA-Pt	Spherical	50.0	5.6	0.0023	410.71	840	93 %	[57]
46	Fe_2O_3-Pt-DSL-Pt	Ellipsoidal	2.0	500	0.0063	12.60	480	100 %	[58]
47	Pt/C	Spherical	1.7	28.5	0.0032	111.11	1080	> 95 %	[59]
**Unsupported Ni-X or X-Pt NPs**									
48	Pd-Pt	Nanotube	≈ 57.0	15	0.0083	555.56	420	≈ 100 %	[49]
49	Ni-Cu	Spherical	15.2	2000	0.0220	11.00	180	100 %	[60]
50	Ni25-Cu75	Spherical	102.0	2000	0.0049	2.45	780	100 %	[61]
51	Au-Pt	Spherical	3.9 ± 1.3	50000	0.1087	2.17	120	100 %	[51]
52	Ni96-Pt4	NR*	19.0 ± 3.0	4	0.0019	483.33	1800	100 %	[52]
53	Pd-Pt	Snowflake	3.8	4	0.0005	131.00	6600	100 %	[62]
54	Ni-Cu-GP	Spherical	68.0	10000	0.0061	610.00	420	97 %	[63]
55	Ag-Pt	Branched	185.47	50	0.0059	118.33	480	> 90 %	[64]
56	Ag-Pt	Spherical	≈ 4	4.53	0.0590	13600	1200	100 %	[65]
57	Au-Pt	NR*	NR*	14.7	0.0016	108.83	2400	≈ 95 %	[65]
**Supported Ni-X or X-Pt NPs**									
58	Cu-Pt/Y	Spherical	6.0	20000	0.0022	0.11	1200	NR*	[66]
59	Ni-Cu-NWs/G	Nanowire	80.0	200	0.0193	96.83	180	98.25 %	[67]
60	Ag-Pt/CeO_2	NR*	15.0-18.0	513	0.0240	46.78	180	100 %	[68]
61	Co-Pt/CNF	Nanofiber	20.0	1000	0.0286	28.60	98	100 %	[69]
62	Ag-Pt/ MWCNTs	Spherical	2.9	5000	0.0108	2.16	1800	≈ 100 %	[70]
63	Co-Pt NDs/N-rGO	Nanodentrite	26.3	15	0.0012	1890	540	≈ 100 %	[71]
64	Cu-Pt-HNT	Nanotube	3.0 ± 0.6	25	0.0062	247	500	≈ 100 %	[72]
65	Cu_3-Pt-HNT	Nanotube	3.1 ± 0.6	20	0.0210	1068	250	≈ 100 %	[72]
66	Cu_3-Pt-HNT-200	Nanotube	2.8 ± 0.4	9.2	0.0110	1190	> 400	≈ 100 %	[72]
67	Cu_3-Pt-HNT-400	Nanotube	2.7 ± 0.6	1.9	0.0228	1222	250	≈ 100 %	[72]
68	$Pt_{0.75}Ni_{0.25}$@Mo2C /NCF	Nanoflowers	1.68 ± 0.6	50	0.0112	224	360	≈ 100 %	[73]
69	Pt–Ni/rHNTs	Nanotubes	1.6	-	0.0033	764.1	900	> 90%	[74]

*TW = This work
*NR = Not Reported

References

25. Vegard, L., The constitution of the mixed crystals and the filling of space of the atoms. *Z Phys* **1921,** *5*, 17-26.
26. Mendoza-Cruz, R.; Bazán-Diaz, L.; Velázquez-Salazar, J.; Samaniego-Benitez, J.; Ascencio-Aguirre, F.; Herrera-Becerra, R.; José-Yacamán, M.; Guisbiers, G., Order–disorder phase transitions in Au–Cu nanocubes: from nano-thermodynamics to synthesis. *Nanoscale* **2017,** *9* (27), 9267-9274.
27. Tarpani, L.; Mencarelli, E.; Nocchetti, M.; Fanò, L.; Taglieri, L.; Latterini, L., Spectrophotometric analysis of nickel colloid performances as catalysts for hydrogenation of nitro-phenol: influence of the stabilizing agents. *Catalysis Communications* **2015,** *15*, 30132-1.
28. Jin, L.; Zhao, X.; Ye, J.; Qian, X.; Dong, M. J. C. C., MOF-derived magnetic Ni-carbon submicrorods for the catalytic reduction of 4-nitrophenol. **2018,** *107*, 43-47.
29. Zhifeng, J.; Jimin, X.; Deli, J.; Xiaojun, W.; Min, C., Modifiers-assisted formation of nickel nanoparticles and their catalytic application to p-nitrophenol reduction. *CrystEngComm* **2013,** *15*, 560-569.
30. Zhifeng, J.; Jimin, X.; Deli, J.; Junjie, J.; Huiru, Q., Facile route fabrication of nano-Ni core mesoporous-silica shell particles with high catalytic activity towards 4-nitrophenol reduction. *CrystEngComm* **2012,** *14*, 4601-4611.
31. Blosi, M.; Albonetti, S.; Costa, A. L.; Sangiorgi, N.; Sanson, A., Easily scalable synthesis of Ni nanosols suitable for the hydrogenation of 4-nitrophenol to p-aminophenol under mild condition. *Chemical Engineering Journal* **2013,** *215-216*, 616-625.
32. Ye, T.; Yanxia, L.; Fu, P.; Fengli, W.; Xiao, Z., Green synthesis of nanostructed Ni-reduced graphene oxide hybridsand their application for catalytic reduction of 4-nitrophenol. *Colloids and Surfaces A: Physicochemical and Engineering Aspects* **2015,** *464*, 96-103.
33. Aili, W.; Hengbo, Y.; Min, R.; Huihong, L.; Jinjuan, X.; Tingshun, J., Preparation of nickel nanoparticles with different sizes and structures and catalytic activity in the hydrogenation of p-nitrophenol. *New Journal of Chemistry* **2010,** *34*, 708-713.
34. Shenghuan, Z.; Shili, G.; Fei, H.; Shujiang, D.; Lei, L.; Piaoping, Y., In situ assembly of well-dispersed Ni nanoparticles on silica nanotubes and excellent catalytic activity in 4-nitrophenol reduction. *Nanoscale* **2014,** *6*, 11181.
35. Nurettin, S.; Hava, O.; Ozgur, O.; Nahit, A., New catalytic route: Hydrogels as templates and reactors for in situ Ni nanoparticle synthesis and usage in the reduction of 2- and 4-nitrophenols. *Applied Catalysis A: General* **2010,** *385*, 201-207.
36. Zhenyuan, J.; Xiaoping, S.; Guoxing, Z.; Hu, Z.; Aihua, Y., Reduced graphene oxide/nickel nanocomposites: facile synthesis, magnetic and catalytic properties. *Journal of Materials Chemistry* **2012,** *2012*, 3471-3477.
37. Tahseen, K.; Abdullah, M. A.; Nauman, A., Catalytic reduction of 4-nitrophenol and methylene blue pollutants in water by copper and nickel nanoparticles decorated polymer sponges. *Spectrochimica Acta Part A: Molecular and Biomolecular Spectroscopy* **2021,** *261*, 120019-120028.
38. Chaoyu, L.; Gan, L.; Huiqin, Z.; Anlin, F.; Li, Z.; Qingyang, L., Ni nanoparticles-loaded ZnO nanowire as an efficient and stable catalyst for reduction of 4-nitrophenol. *EcoMat* **2022,** *4*, 12164.
39. De Bin, J.; Xiaoying, L.; Yunsong, Y.; Li, F.; Junyi, J.; Jinshu, W.; Dusan, L.; Hong-Chang, Y.; Yu, X. Z., Biotemplated top-down assembly of hybrid Ni nanoparticles/N doping carbon on diatomite for enhanced catalytic reduction of 4-nitrophenol. *Chemical Engineering Journal* **2020,** *383*, 123156.
40. Jianping, W.; Min, Z.; Teng, M.; Yang, L.; Qiong, W.; Jing, Z.; Jingli, X.; Tasawar, H.; Njud, S. A., A facile self-template and carbonization strategy to fabricate nickel nanoparticles supported N-doped carbon microtubes. *Inorganic Chemistry Frontiers* **2018,** *5* (4), 844-852.
41. Ding, L.; Zhang, M.; Zhang, Y.; Yang, J.; Zheng, J.; Xu, J., A type of raspberry-like silica composite with tunable nickel nanoparticles coverage towards nanocatalysis and protein adsorption. *Green Chemistry* **2016,** *18* (23), 6282-6290.
42. Wang, N.; Wen, Q.; Liu, L.; Xu, J.; Zheng, J.; Yue, M.; Asiri, A. M.; Marwani, H. M.; Zhang, M., One dimensional hierarchical nanoflakes with nickel-immobilization for high performance catalysis and histidine-rich protein adsorption. *Dalton Transactions* **2019,** *48*, 11308.
43. Ding, L.; Zhang, M.; Zheng, J.; Xu, J.; Alharbi, N. S.; Wakeel, M., Fabrication of Ultrafine Nickel Nanoparticles Anchoring Carbon Fabric Composites and Their High Catalytic Performance. *Colloids and Surfaces A: Physicochem. Eng. Aspects* **2019,** *562*, 146-153.

44. Bogireddy, R. N. K.; Pal, U.; Kumar, M. K.; Domínguez, J. M.; Martínez, G. L.; Agarwal, V., Green fabrication of 2D platinum superstructures and their high catalytic activity for mitigation of organic pollutants. *Catalysis Today* **2021,** *360*, 185-193.
45. Jyun-Guo, Y.; Chandirasekar, S.; Yao-Wen, L.; Cheng-Ju, Y.; Wei-Lung, T., Boosting Catalytic Activity of Metal Nanoparticles for 4-Nitrophenol Reduction: Modification of Metal Naoparticles with Poly(diallyldimethylammonium chloride). *Journal of Hazardous Materials* **2017,** *324*, 420-427.
46. Mourdikoudis, S.; Altantzis, T.; Liz-Marzán, L. M.; Bals, S.; Pastoriza-Santos, I.; Pérez-Juste, J., Hydrophilic Pt nanoflowers: synthesis, crystallographic analysis and catalytic performance. *CrystEngComm* **2016,** *18*, 3422.
47. Pandey, S.; Mishra, S. B., Catalytic reduction of p-nitrophenol by using platinum nanoparticlesstabilised by guar gum. *Carbohydrate Polymers* **2014,** *113*, 252-531.
48. Ullah, S.; Ahmad, A.; Wang, A.; Raza, M.; Ullah, J. A.; Tahir, K.; Rahman, A. U.; Qipeng, Y., Bio-fabrication of catalytic platinum nanoparticles and their in vitro efficacy against lungs cancer cells line (A549). *Journal of Photochemistry & Photobiology, B: Biology* **2017,** *173*, 368-375.
49. Wang, Y.; Li, Q.; Zhang, P.; O'Connor, D.; Varma, R. S.; Yu, M.; Hou, D., One-pot Green Synthesis of Bimetallic Hollow Palladium-platinum Nanotubes for Enhanced Catalytic Reduction of p-Nitrophenol. *Journal of Colloid and Interface Science* **2019,** *539*, 161-167.
50. Ramírez-Crscencio, F.; Redón, R.; Herrera-Gómez, A.; Gómez-Sosa, G.; Bravo-Sánchez, M.; Fernández-Osorio, A. L., Facile obtaining of Iridium(0), Platinum(0) and Platinum(0)-Iridium(0) alloy nanoparticles and the catalytic reduction of 4-nitrophenol. *Materials Chemistry and Physics* **2017,** *201*, 289-296.
51. Zhang, J.; Chen, G.; Guay, D.; Chaker, M.; Dongling, M., Highly active PtAu alloy nanoparticle catalysts for the reduction of 4-nitrophenol. *Nanoscale* **2014,** *6*, 2125.
52. Ghosh, S. K.; Mandal, M.; Kundu, S.; Nath, S.; Pal, T., Bimetallic Pt–Ni nanoparticles can catalyze reduction of aromatic nitro compounds by sodium borohydride in aqueous solution. *Applied Catalysis A: General* **2004,** *268*, 61-66.
53. Qianli, W.; Yiwei, Z.; Yuming, Z.; Zewu, Z.; Jinjuan, X.; Yuanmei, X.; Chao, Z.; Xiaoli, S.; Naishu, K., Nanocasting synthesis of an ordered mesoporous CeO2-supported Pt nanocatalyst with enhanced catalytic performance for the reduction of 4-nitrophenol. *RSC Advances* **2016,** *6*, 730-739.
54. Zhang, P.; Yang, X.; Peng, H.; Liu, D.; Lu, H.; Wei, J.; Gui, J., Magnetically Recoverable Hierarchical Pt/Fe2O3 Microflower: Superior Catalytic Activity and Stability for Reduction of 4-Nitrophenol. *Catalysis Communications* **2017,** *100*, 214-218.
55. Reddy, B. N. K.; Sahare, P.; Pal, U.; Olive, M. S. F.; Martínez, G. L.; Agarwal, V., Platinum nanoparticle-assembled porous biogenic silica 3D hybrid structures with outstanding 4-nitrophenol degradation performance. *Chemical Engineering Journal* **2020,** *388*, 124237.
56. Shouliang, W.; Jun, L.; Yixing, Y.; Zhenfei, T.; Pengfei, L.; Yunyu, C.; Yue, L.; Changhao, L., In-situ reactive loading of platinum onto tin oxide nanocrystals with superior catalytic performance for hydrogenation of 4-nitrophenol. *Applied Surface Science* **2019,** *471*, 469-474.
57. Shao-Wei, B.; Si, L.; Lan, C., Synthesis of magnetically recyclable Fe3O4@polydopamine–Pt composites and their application in hydrogenation reactions. *Journal of Materials Science* **2016,** *51*, 3643-3649.
58. Yan, G.; Jiasheng, F.; Yiwei, Z.; Chao, Z.; Shuo, Z.; Yuming, Z.; Mengqiu, H.; Xiaoli, S., Novel synthesis of Fe2O3–Pt ellipsoids coated by double-shelled La2O3 as a catalyst for the reduction of 4-nitrophenol. *Applied Organometallic Chemistry* **2018,** *32* (3), 4208.
59. Wenjun, G.; Siwen, L.; Manas, P.; Yong, L.; Xiaoyue, W.; Wei, L.; Shuai, W.; Changyao, W.; Gengfeng, Z.; Dongyuan, Z., Capping Agent-Free Highly Dispersed Noble Metal Nanoparticles Supported in Ordered Mesoporous Carbon with Short Channels and Their Catalytic Applications. *RSC Advances* **2016,** *6* (66), 61064-61072.
60. Seethapathy, V.; Sudarsan, P.; Pandey, A. K.; Pandiyan, A.; Kumar, T. H. V.; Sanjeevi, K.; Sundramoorthy, A. K.; Moorthy, S. B. K., Synergistic effect of Bimetallic Cu:Ni nanoparticles for the efficient catalytic conversion of 4 –Nitrophenol. *New Journal of Chemistry* **2019,** *43* (7), 3180-3187.
61. Vivek, S.; Preethi, S.; Vignesh, K. T. H.; Sundramoorthy, A. K.; Suresh, B. K., Oxidation studies on mono (Cu, Ni) and bimetallic (Cu–Ni) nanoparticles and its impact on catalytic activity. *Journal of Alloys and Compounds* **2020,** *816*, 152608.
62. Ya, T.; Guangfei, L.; Dong, B.; Huali, Y.; Jiti, Z.; Jing, W.; Ruofei, J., Microbial synthesis of bimetallic PdPt nanoparticles for catalytic reduction of 4-nitrophenol. *Environ Sci Pollut Res* **2017,** *24*, 5249-5258.

63. Ismail, M.; Khan, M. I.; Khan, S. B.; Khan, M. A.; Akhtar, K.; Asiri, A. M., Green synthesis of plant supported Cu\\Ag and Cu\\Ni bimetallic nanoparticles in the reduction of nitrophenols and organic dyes for water treatment. *Journal of Molecular Liquids* **2018,** *260*, 78-91.
64. Zhi-Suo, L.; Xiao-Yan, Z.; Han-Bin, M.; Jiu-Ju, F.; Ai-Jun, W., One-pot synthesis of highly branched Pt@Ag core-shell nanoparticles as a recyclable catalyst with dramatically boosting the catalytic performance for 4-nitrophenol reduction. *Journal of Colloid and Interface Science* **2019,** *538*, 349-356.
65. Varshney, S.; Bar-Ziv, R.; Zidki, T., On the Remarkable Performance of Silver-based Alloy Nanoparticles in 4-nitrophenol Catalytic Reduction. *ChemCatChem* **2020,** *12* (18), 4680-4688.
66. El-Bahy, Z. M.; Hanafy, A. I.; El-Bahy, S. M., Preparation of Pt, Pd and Cu nano single and bimetallic systems-supported NaY zeolite and test their activity in p-nitrophenol reduction and as anticancer agents. *Journal of Environmental Chemical Engineering* **2019,** *7*, 103117.
67. Zhiqiang, X.; Xianhua, H.; Mingwei, L.; Lijuan, S.; Di, L.; Kenan, X.; Li, L., Catalytic reduction of 4-nitrophenol over graphene supported Cu@Ni bimetallic nanowires. *Materials Chemistry and Physics* **2019,** *227*, 64-71.
68. Kharlamova, T. S.; Salina, M. V.; Svetlichnyi, V. A.; Salaev, M. A.; Stadnichenko, A. I.; Mamontov, G. V., CeO2-supported Pt–Ag bimetallic catalysts for 4-nitrophenol reduction. *Catalysis Today* **2022,** *384-386*, 12-24.
69. Langde, V. K.; Hakke, V. S.; Kakunuri, M.; Babu, G. U. B.; Boczkaj, G.; Sonawane, S. H., Synthesis of bimetallic Co–Pt/cellulose nanocomposites for catalytic reduction of p-nitrophenol. *Reaction Chemistry & Engineering* **2022,** *7*, 641.
70. Esquivel-Peña, V.; Bastos-Arrieta, J.; Muñoz, M.; Mora-Tamez, L.; Munguía-Acevedo, N. M.; Ocampo, A. L.; De Gyves, J., Metal nanoparticle–carbon nanotubes hybrid catalysts immobilized in a polymeric membrane for the reduction of 4-nitrophenol. *SN Applied Sciences* **2019,** *1*, 347.
71. Xiao-Fang, Z.; Xiao-Yan, Z.; Jiu-Ju, F.; Ai-Jun, W., Solvothermal synthesis of N-doped graphene supported PtCo nanodendrites with highly catalytic activity for 4-nitrophenol reduction. *Applied Surface Science* **2018,** *428*, 798-808.
72. Xiaobin, G.; Feng, T.; Zhaoxia, J., Pt−Cu Bimetallic Nanoparticles Loaded in the Lumen of Halloysite Nanotubes. *Langmuir* **2019,** *35*, 14651-14658.
73. Shanmugaraj, K.; Manikandan, V.; Campos, C. H.; Vinoth, V.; Mangalaraja, R. V.; Mathivanan, M.; Pugazhenthiran, N.; de León, J. N. D.; Song, K. S., A highly efficient and stable PtNi bimetallic nanoparticles modified on Mo2C decorated N-doped carbon flowers catalysts for the remediation of environmental pollutants. *Journal of Environmental Chemical Engineering* **2024,** *12* (5), 113948.
74. Duan, J.; Zhao, Y.; Zhai, Z.; Chen, S.; Zhang, B., Decoration of Pt–Ni Alloy on Molten Salt Etched Halloysite Nanotubes for Enhanced Catalytic Reduction of 4-Nitrophenol. *Separations* **2024,** *11* (11), 305.
